# Revealing detrimental effects of various DC electrical energy conditions on different multidrug resistant bacteria: a comprehensive study

**DOI:** 10.1038/s41598-024-66063-4

**Published:** 2024-07-24

**Authors:** Mamdouh M. Shawki, Hadeel S. El-Shall, Maisa E. Moustafa, Kamal Y. S. Atay, Amel G. Elsheredy, Marwa M. Eltarahony

**Affiliations:** 1 Medical Biophysics Department, Medical Research Institute, Alexandria University, Alexandria, Egypt; 2 Environmental Biotechnology Department, Genetic Engineering and Biotechnology Research Institute, City of Scientific Research and Technological Applications (SRTA-City), Alexandria, Egypt; 3 Microbiology Department, Medical Research Institute, Alexandria University, Alexandria, Egypt

**Keywords:** Biophysics, Biotechnology, Microbiology, Environmental sciences, Environmental social sciences, Medical research, Physics

## Abstract

The arbitrary discharge of contaminated wastes, especially that encompass multidrug resistant microbes (MDR), would broaden the circle of epidemic diseases such as COVID-19, which in turn deteriorate definitely the whole socioeconomics. Therefore, the employment of electrical stimulation techniques such as direct current (DC) with low energy considers being effective tool to impede spontaneous changes in microbial genetic makeup, which increases the prevalence of MDR phenomenon. Herein, the influence of different electric energies generated by DC electric field, volts and time on MDR-bacteria that are categorized among the highly ranked nosocomial pathogens, was scrutinized. Wherein, *Pseudomonas aeruginosa, Escherichia coli, Staphylococcus aureus* and *Enterococcus faecalis* were examined as paradigms of Gram-negative and Gram-positive pathogens. The results declared the significant superior antagonizing potency of electric energy in a dose-dependent modality rather than the applied volts or exposure time. Notably, the exposure of bacterial cultures to140 J inhibited the bacterial count by > 78% and the range of 47–73% for Gram-negative and Gram-positive, respectively. While the suppression in their metabolic activity assessed by > 75% and 41–68%, respectively; reflecting the capability of electrical energy to induce viable but non-culturable (VBNC) state. Similarly, the results of total protein, extracellular protein content and lactate dehydrogenase activity emphasized the cell wall deterioration and losing of cell membrane integrity. Additionally, the elevating in ROS upon DC-exposure participated in DNA fragmentation and plasmid decomposability by the range of 33–60%. Further, SEM micrographs depicted drastic morphological deformations after electrical treatment. Strikingly, DC-treatment impaired antibiotic resistance of the examined strains against several antibiotics by > 64.2%. Generally, our comparative detailed study revealed deleterious potentiality of different DC-protocols in defeating microbial pollution, which could be invested as efficient disinfectant alternative in various sectors such as milk sterilization and wastewater purification.

## Introduction

Infection is recognized as the assaulting of the host by microorganisms, which thereafter rapidly multiply in the host's tissues. The pathogenicity of such microbes manifested clearly through their capacity to cause diseases. Based on the microbial pathogenicity, the microbes could be classified as frank/virulent, opportunistic-pathogen or avirulent/nonpathogenic. As referred by World Health Organization (WHO) reports, infectious diseases caused by microbial pathogens are the substantial reason for increasing the mortality and morbidity rates^1^. Importantly, such microbial infections could be disseminated worldwide through multiple pathways including foodborne, waterborne and nosocomial pathogens. Wherein, wastewater/drinking water purification systems, food manufacturing, processing, storage, packaging, livestock feces, slaughtering houses effluents, surgical equipment, hospital surfaces, medical devices, health care utilities and hygienic products symbolize the most common routs. Remarkably, the most serious issue in this pathogenic infections/ injuries is the prevalence of Multidrug-Resistance infections (MDR), which deemed as environmental dilemma and global public health challenge that has been increasing in severity and scope^2^.

However, the uncontrolled discharge of any contaminated wastes from previously mentioned sources, especially which contain MDR-microbes, into the surrounding environment (i.e. soil and water) would definitely expand the circle of epidemic diseases and deteriorate the whole ecosystem^1,3^. With the progressive outbreak and continuous mutation of COVID-19 and its conjugate bacterial-post infections, the safe and effective treatment of wastes is crucial to protect public health, social security and also economy. Several techniques are continuously developed to prevent or at least reduce the spread of MDR-microbes such as chlorination, ultraviolet, ozonation and hydrogen peroxide disinfection. Despite their effectiveness, each technique suffers from several limitations regarding to ecological environment safety as reported by^4^. Let alone the persistence of microbes to overcome the effect of any antimicrobial agents by different strategies such as spontaneous changes in their genetic makeup, alteration of active sites by enzymatic modification, sequestering the disinfectant molecule and enhancing active efflux^5^. As a consequence, it is imperative to overcome such stalemate through recent technological practices with antimicrobial effectiveness. Interestingly, electrical stimulation techniques (e.g., direct current (DC), low-intensity direct current (LIDC), alternating current (AC)) exhibit imminent contribution in defeating bacteria either as bactericidal or bacteriostatic activity as reported by^1^. Additionally, several reports documented the effect of polarity and current intensity in wound healing by inhibiting the bacterial growth associated with wounds/burns^1,6^.

It is plausible to mention that the electrified processes (EF) are emerging and have become promising treatment-alternatives due to the low cost of the used electricity, and independence of chemicals. EF is a physical pathogen inactivation approach, which has been studied for both water disinfection and food pasteurization^7^. It has shown great potential in processing liquid food such as juice, alcoholic, and dairy products. As a non-thermal process, it will not affect the food's flavor, texture, and nutrients if the processing temperature is controlled. EF systems available on the market can process up to 10,000 L of liquid food per hour. The cost for the pathogen inactivation of beverages is estimated to be 10 Euro/ton, which is already affordable in some circumstances^8^

Strikingly, the electric current modality in blocking the bacterial growth could be exerted either by damaging the bacterial membrane directly or preventing the division of bacterial cells, which results in cell death. Also, biocides are produced during the electrochemical process, which causes unfavorable changes in the composition of bacteria that ultimately result in their death^9^. Galvanotaxy, which is the migration of particles to a cathode or anode under the influences of an electric field, induces possible change in temperature /pH, and creates of hazardous electrolytic byproducts like H_2_O_2_ and oxidizing radicals. Via such method, the electric current influences indirectly on bacterial mortality^10,11^. Notably, as recorded by^12^, weak electric fields have been demonstrated to improve the effectiveness of antibiotics. Wherein, the concentrations of antibiotics required to be effective against biofilm bacteria decreased to only 1.5 to 4.0 times comparing to those required for planktonic bacteria with the application of direct current electric fields of approximately 1.5 to 20 V/cm, which is known as the bioelectric effect^13^. Low voltage direct-current (DC) also controls the biocidal concentrations of some metals. Previously, a coaxial-electrode copper ionization cell was developed to combine copper disinfection with locally enhanced electric field treatment, demonstrating superior disinfection efficiency with low effluent copper concentrations (< 0.5 mg/L). However, using DC voltages lead to a dilemma that a higher voltage is necessary for effective electric disinfection but a lower voltage is required to limit copper release^14^.

Accordingly, the current study aimed to evaluate the bactericidal effect of DC electric field on MDR-bacterial strains affiliated to both Gram-positive and Gram-negative types, in detailed and comparative manner. Also, to find if such DC effect could change the bacterial sensitivity to different antibiotics. That would be implemented via applying electric energy in two different protocols of volts and exposure time. One protocol was to examine the effect of different electrical energies at constant exposure time and the other protocol was to examine different exposure times with the same electrical energy.

## Materials and methods

### Preparation and growth conditions of the examined pathogens

The following bacterial strains were utilized in the current investigation: *P. aeruginosa* (ATCC27853) and *E. coli* (ATCC25922) as Gram-negative strains, on the other hand, *S. aureus* (ATCC25923), and *E. faecalis* (ATCC29212) as Gram-positive types. They were cultured on blood agar plates and incubated at 37 °C under aerobic conditions for 16–24 h.

### Exposure system

The bacterial culture of each examined strain was prepared by inoculating an overnight loopful of the culture into 100 mL of LB media with the following components (g/L): tryptone 10, yeast extract 5, NaCl 10, and pH adjusted to be 7.0. After 24 h incubation in a rotary shaker incubator (150 rpm) at 37 °C, the bacterial suspensions with bacterial count assessed by ~ 1 × 10^9^ CFU/ mL were exposed to DC electric field in a conical sterile polypropylene tube by placing the bacterial suspension between two rectangular Ag/AgCl electrodes. The electrodes were connected to a DC power supply (Etommens eTM-305A, made in China) of voltage application range from 0.1 to 28 V.

### Experiment design

The electrical resistance of the bacteria in the media was measured as 120 ± 5 Ω. The electrical energy (E) in J can be calculated as:

1 $${\text{E}} = {\text{V}} \cdot {\text{I}} \cdot {\text{T}}$$

where V is the potential difference (v), I is the electrical current (A), and t is the exposure time (s).

To determine the lethal electrical energy that inhibited the bacterial growth completely, and to indicate the main electrical factor that led to bacterial death, either electrical energy or electrical parameters (electrical voltage and current), the following pilot study was conducted including two exposure protocols.

The first exposure protocol aimed to examine the effect of increasing applied electrical energy on each bacterial strain (A1 to A6) at a fixed exposure time of 300 s by changing the applied voltage. The Groups were exposed to 15 J, 60 J, 240 J, 562.5 J, 1020 J, and 1500 J corresponding to groups A1-A6, respectively. The second exposure protocol aimed to examine the effect of changing the applied voltage and the exposure time on each bacterial strain (B1 to B6) with the generation of 300 J as a fixed exposure electrical energy. The groups were exposed to 2.5 V for 6000 s, 5 V for 1500 s, 10 V for 375 s, 15 V for 167 s, 20 V for 94 s, and 25 V for 60 s corresponding to groups B1–B6, respectively.

The main experiment was then conducted based on the results of the performed optimization experiment; four sub-lethal electrical conditions were determined to be applied against tested bacterial species. The importance of this step lied behind clarifying the concept of the determinant effective factor, which is the applied total electrical energy. Therefore, the used electrical parameters as follows:

- Group 1: Applied voltage is 5 V for 300 s with a total produced energy of 60 J.
- Group 2: Applied voltage is 2.5 V for 1200 s with a total produced energy of 60 J.
- Group 3: Applied voltage is 7.5 V for 300 s with a total produced energy of 140 J.
- Group 4: Applied voltage is 2.5 V for 2760 s with a total produced energy of 140 J.

After exposure, the following parameters have been measured to determine each electric group's effect on the examined bacterial species as follows.

### Heat production measurement, galvanic reaction observation and released ions determination

The temperature of each bacterial suspension was measured just before and just after the electric exposure by a digital thermometer (GRANZIA, made in China). Also, observation of the electrodes and the media color change before and after the exposure were noticed and recorded to indicate the galvanic reaction and media change due to DC exposure. Subsequently, silver ions that were released from the electrodes during exposure protocols were determined quantitatively using Inductive Coupled Plasma Mass Spectrometry (ICP) (Agilent ICP-OES 5110DVD).

### Cultivability assessment and determination of metabolic activity

The bacterial count (CFU/mL) for each examined strain was determined via pour plate method. About 1 mL of bacterial suspensions, either treated and untreated samples, were serially diluted and poured into sterile petri dish, followed by addition of molten agar (40–50 °C) and swirling several times clockwise and anticlockwise. After solidification, the plates were incubated at 37 °C for 24 h. The viable cells forming colonies were counted after complete incubation and the growth inhibition percentage was calculated according to the equation reported in^15^.

2 $${\text{Growth}} \;{\text{inhibition}}\;{\text{percentage}} = \frac{{{\text{Number}}\;{\text{of}}\;{\text{colonies}}\;{\text{in}}\;{\text{control}} {-}{\text{ Number}}\;{\text{of}}\;{\text{colonies}}\;{\text{after}}\;{\text{treatment}}}}{{{\text{Number}}\; {\text{of}}\;{\text{colonies}}\; {\text{in }}\,{\text{control}}}} \times 100$$

The metabolic performance of any viable cell is determined calorimetrically using MTT assay. About 5 mg/mL of (3-[4,5-dimethylthiazol-2-yl]-2,5-diphenyltetrazolium bromide powder was dissolved in sterile distilled water at room temperature then filtered through 0.22 μm Whatman filter paper. In the presence of metabolically active bacterial cells, MTT is reduced to purple formazan. Briefly, 40 mL nutrient broth medium containing 20 μL of 10^8^ cell/mL bacterial strains exposed to the exposure protocols. Control unexposed bacterial cells were used as the blank control. All flasks were incubated with shaking at 37 °C at 200×*g* for 24 h; then 1 mL from each flask containing the treated and the control cultures was added to sterilized test tubes containing 100 μL MTT solution (0.5%w/v). All tubes were incubated at 37 °C for 1 h. The resulting formazan was centrifuged at 4000×*g* for 3 min followed by decantation of the supernatants. The pellets obtained were resuspended and centrifuged again in DMSO. The purple formazan solution obtained at the end, which indicates the activity and viability of the cells was measured by spectrophotometer at 570 nm^16^.

### Determination of total and extracellular protein content

Total cellular protein and extracellular protein concentrations were determined by Bradford assay through using of a commercially colorimetric protein assay kit according to the manufacturer protocol (Bio-Rad) with bovine serum albumin (BSA) as a standard^17^). Different volumes (10 µL, 20 µL, 40 µL, 60 µL, 80 µL, and 100 µL) of working standard were pipette out into a series of test tubes. 100 µL of supernatant from each flask was pipette out into other tubes. The volume of the tubes was made up to 1 mL using sterilized distilled water. 5 mL of the Bradford reagent was added to all the tubes and mixed thoroughly. 1 mL of distilled water with 5 mL of Bradford reagent was used as blank. Absorbance at 595 nm was recorded against blank.

### Determination of lactate dehydrogenase (LDH) activity

The cell membrane instability for all treated and control samples was studied via determining LDH activity^18^. 100 μL of the supernatant from each microbial culture treated with different exposure protocol was added to the reaction mixture containing 0.5 mL of 100 mM pyruvate, 5 mg NADH in 20 mL of 500 mM potassium phosphate buffer, and pH 7.5 at 37 °C. Absorbance (A) was recorded for 0.5 to 5 min and relative change in the absorbance per minute (Δ*A*/min) was calculated at 340 nm using UV–visible spectrophotometer. LDH activity was expressed in (U/L) which is the amount of enzyme that reduces 1 μM of NAD per min. at a specific temperature:

3 $${\text{U}}/{\text{L}} = (\Delta A/\min \times {\text{TV}} \times 1000/{\text{d}}) \times \varepsilon \times {\text{SV}}$$

where TV is the total reaction volume, 1000 is the conversion of U/mL into U/L, *d* is the light path in cm, ε is the absorptivity of NADH in mM, and SV is the sample volume in mL.

### Determination of total DNA

The genomic material of treated and untreated control samples was prepared from overnight cultures using AMSHAG—DNA Extraction Kit^19^. Briefly, to each of overnight fresh culture, an aliquot of 1 mL of AMSHAGE DNA extraction kit was added. The tubes were mixed thoroughly, inverted gently several times till complete re-suspension and incubated in a water bath at 70 °C for 30 min. After complete cell lysis, the tubes were centrifuged for 15 min at 12,000 rpm. The upper 500 µL of the supernatant was transferred to a new tube containing 500 µL of ice cold isopropanol, incubated for 10 min at room temperature, thereafter the mix was centrifuged for 15 min at 14,000 rpm. The supernatant was then gently decanted. Pellets were washed two times with 70% ethanol, air dried and re-suspended in 50 µL sterile water. Volumes of 5 µL of the extracted DNA were examined by loading on 0.8% agarose gel (FMC, Rockland, USA) containing ethidium bromide at 0.1 µg/mL. Gels were run at 100 V in 1X TBE buffer and then visualized and photographed in the MultiImage light cabinet. 1 Kb ladder mix (GeneRuler™ Fermentase) was used as a molecular weight marker. For quantitative determination of DNA concentration in both control and treated cells, the absorbance of 5 µL of the extracted DNA was measured at 260 nm (OD260) by a UV–VIS spectrophotometer^20^.

### Determination of reactive oxygen species (ROS)

The ROS generated following the different exposure protocols were determined by FDA, 3,6-diacetoxyfluoran assay^21^. 100 μL of FDA (10 μg/mL) was added to each treated sample and control and incubated for 30 min at 37 °C. After incubation, the cleavage of FDA was stopped by the addition of acetone to a final concentration of 50% v/v. To eliminate suspended particles, the mixture was centrifuged for 5 min at 10,000 rpm. The fluorescence intensity was analyzed by a fluorimeter microplate reader (FluoStar Omega, Germany) with excitation and an emission wavelength at 495 nm and 525 nm, respectively. The concentration of ROS was determined by the standard curve of H_2_O_2_ at different concentrations.

### Determination of ultrastructure changes

The morphological changes of each bacterial strain after exposure protocol, compared to untreated control cells, were visualized by scanning electron microscope (SEM). Initially, 3% glutaraldehyde in phosphate buffer serum (PBS) was used for 12 h to fix bacterial cell. Thereafter, the bacterial cultures were washed with 4% OsO_4_ in 0.1 M phosphate buffer for 2 h, followed by a dehydration step in ethanol gradient (25%, 50%, 75%, and 100%). The samples were examined by SEM (JEOL JSM 6360LA, Japan) after the gold coating step. Samples were then observed at an accelerating voltage of 20 kV.

### Determination of antibiotic sensitivity

The susceptibility of each examined bacterial suspensions after differently applied treatments, in comparison to the control group, were determined by the Kirby Bauer technique (disc diffusion) assay^22,23^. The tested antibiotics were selected according to the Clinical and Laboratory Standards Institute (CLSI) recommendations 2021. All antibiotic discs were provided by Oxoid® and stored in the refrigerator at 2–8 °C. Müller-Hinton Agar was prepared from a dehydrated base according to the manufacturer’s instructions. For diagnosis of antibiotic susceptibility of *P. aeruginosa*; the following antibiotic discs were used: Ceftazidine (CAZ), Gentamycin (CN), Pipadlin (TPZ), Ciprofloxacin (CIP), Norfloxacin (NOR), Amikacin (AK), Cefepime (FEP), and Imipenem (IPM). For* E.coli*: Ampicilin (AMP), Gentamycin (CN), Meropenem (MER), Norfloxacin (NOR), Ciprofloxacin (CIP), Ceftazidine (CAZ), Ofloxacin (OFX), and Tigecycline (TGE). For *S. aureus*: Ciprofloxacin (CIP), Ofloxacin (OFX), Gentamycin (CN), Meropenem (MEM), Rifampin (RD), Erythromycin (E), Vancomycin (VA), and Norfloxacin (NOR). For *E. faecalis*: Ampicilin (AMP), Vancomycin (VA), Ciprofloxacin (CIP), Norfloxacin (NOR), Nitrofurantoin (F), Rifampin (RD), Erythromycin (E), Doxycycline (DO).

To standardize the inoculum density for a susceptibility test, a 0.5 McFarland (10^8^ CFU/mL) was used. A sterile cotton swab was dipped into the adjusted suspension, rotated several times, and pressed firmly on the inside wall of the tube above the fluid level. The dried surface of a Müller-Hinton agar was inoculated by streaking the swab over the entire sterile agar surface. The antimicrobial discs were dispensed onto the surface of the inoculated agar plate. Each disc was pressed down to ensure complete contact with the agar surface. They were distributed evenly and no closer than 24 mm from the center. The plates were inverted and placed in an incubator at 37ºC for 16–18 h. After complete incubation period, each plate was inspected for inhibition zone. The diameters of the complete inhibition zones (as judged by the unaided eye) were measured, including the diameter of the disc using a ruler, which was held on the back of the inverted Petri plate. The Petri plate was held a few inches above a black, non-reflecting background and illuminated with reflected light. Resistant (R) means no zone of inhibition at all^24^.

### Statistical analysis of the data

All data were expressed as mean ± standard deviation (M ±SD) for three independent repeats. ANOVA was used to evaluate the difference between multiple groups. Data were described and analyzed using Graphpad Instat software. Results were considered statistically significant when the p-value ≤ 0.05.

## Results

### Optimization of electrical conditions

#### Determination of DC electric energy

**Figure 1:**
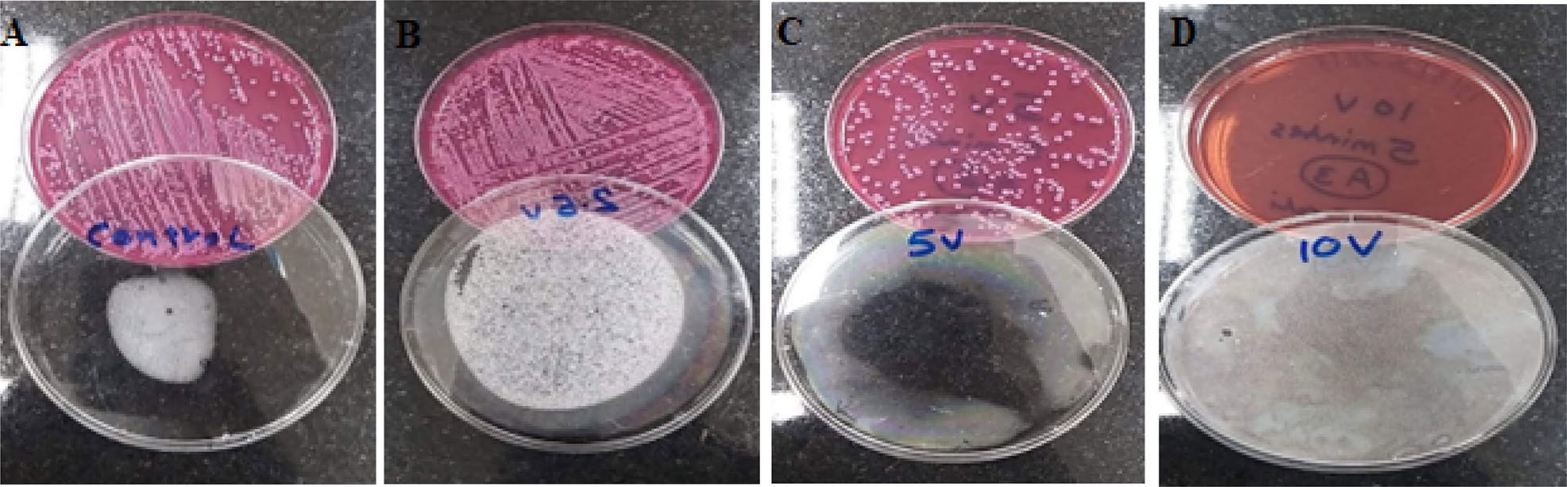
Different applied electrical energies with voltages of 2.5–10 v at a fixed exposure time of 300 s on *E. coli*: (**A**) Control, (**B**) 2.5 V (15 J), (**C**) 5 V (60 J), and (**D**) 10 V (240 J).

As a first step in the screening procedure, the electrical energy that entirely inhibited the bacterial growth was determined via applying different potential differences of 2.5 v to 25 v at a fixed exposure time of 300 s, with applied energies ranged from 15 to 1500 J. The results showed that the cell viability reduction was dose dependent. Wherein, 240 J (10 V for 300 s) resulted in full absence of bacterial growth for all the tested bacteria, while less than 240 J considered as a sub-lethal dose. Figure 1 symbolizes a representative example of such effect on *E. coli*.

#### Determination of DC voltage and exposure time.

To emphasize the results of the first step and also to examine the bactericidal effect of the same produced electrical energy as a function of electrical voltage and time of exposure, the second stage was performed at a fixed applied electrical energy of 300 J (energy dose that caused 100% cell inhibition for all the examined bacterial strains as determined by the first step). The electrical energies were implemented by applying different combinations of voltages and times of exposure as: 2.5 V for 6000 s, 5 V for 1500 s, 10 V for 375 s, 15 V for 167 s, 20 V for 94 s, and 25 V for 60 s. The results showed complete cell inhibition for all the tested bacterial types at 300 J despite the used condition.

### Selection of the exposure electrical conditions

**Figure 2:**
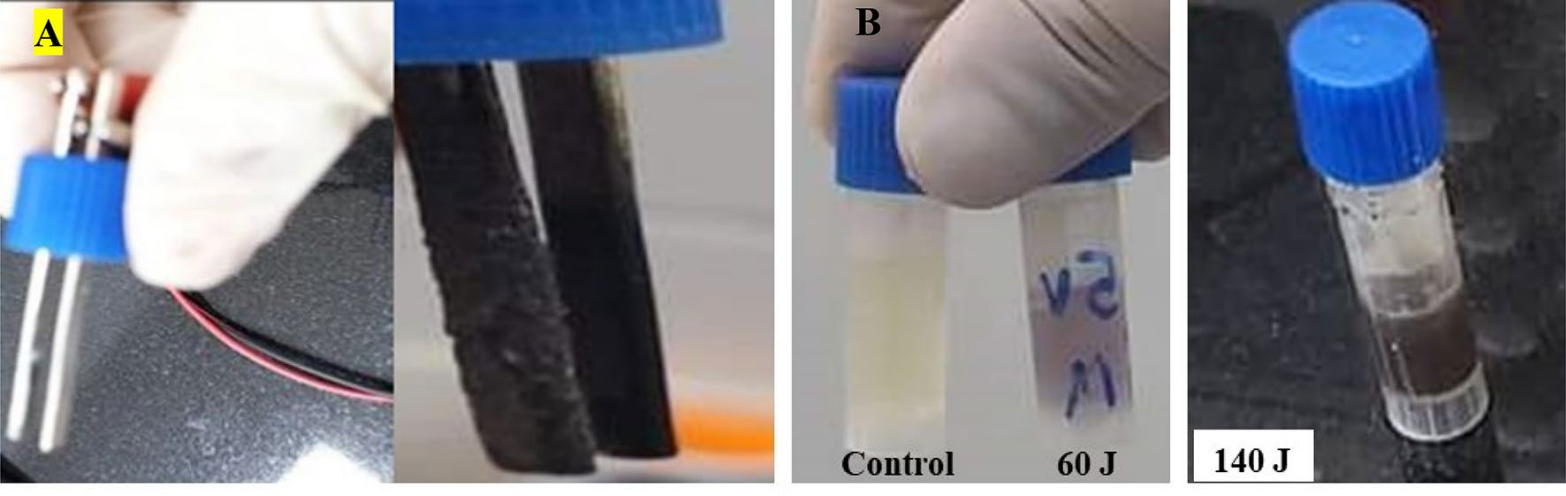
Effect of the applied electric electric energy on the (**A**) galvanic reaction and (**B**) media color.

Based on the optimization results, the decisive factor in suppressing the bacterial growth, either electrical energy or electrical parameters (electrical voltage and current), was defined. Thus, four sub-lethal electrical conditions were applied, with varying both voltage and time simultaneously as a function of electrical energy, namely 60 J and 140 J. Thereafter, the heat production and galvanic reaction generated after each exposure condition in bacterial cultures were determined. As observed, the produced heat due to ohmic heating, when DC was passing through a medium, was directly proportional to the applied electrical energy. Namely, there was an increase in the bacterial suspension temperature by 2.1 ± 0.2 °C and 4.7 ± 0.4 °C in 60 J and 140 J groups, respectively. Both temperatures increment cannot be the main reason for bacterial death as the bacteria can still be viable and active at such temperature elevation. On the other hand, there was a very obvious galvanic reaction in the used electrodes as shown in Fig. 2A, indicating the release of the electrode ions, which led to changing in media color. Remarkably, the change in the media color increased by increasing the applied energy as shown in Fig. 2B, which in turn proposed to uplift ROS production and subsequently adverse impact on bacterial survival.

### The effect of different exposure conditions on examined pathogens:

After exposure, the following parameters were measured to determine each electric group's effect on the examined bacterial species.

#### Cultivability assessment

The inhibitory potency of applied electrical protocols was scrutinized against different genera, which was implemented by determining their count before and after exposure (Table 1). Generally, the inhibition potency could be described as energy dependent, which is clearly significant between 60 and 140 J groups. However, it was insignificant within the same energy group (i.e., between (5 V for 300 s) and (2.5 V—1200 s) of 60 J-group and (7.5 V—300 s) and (2.5 V—2760 s) of 140 J), as revealed by ANOVA. Wherein, the lowest suppression effect was recorded in groups of 60 J, in particular that applied at 5 V for 300 s, this effect is an extremely statistically significant reduction (*P* < 0.0001) compared to control groups. The bacterial count recorded 1.77 × 10^9^ ± 0.1 × 10^9^, 2.71 × 10^9^ ± 0.10 × 10^9^, 2.82 × 10^9^ ± 0.24 × 10^9^, and 4.71 × 10^9^ ± 0.13 × 10^9^ CFU/mL for *P. aeruginosa, E. coli, S. aureus* and *E. faecalis* respectively. Notably, the maximum and significant reduction in bacterial count was observed upon elevating the applied electrical energy, particularly at 140 J (2.5 V for 2760 s). Wherein, the bacterial count enumerated as 1.09 × 10^9^ ± 0.06 × 10^9^, 1.83 × 10^9^ ± 0.07 × 10^9^, 1.53 × 10^9^ ± 0.13 × 10^9^, and 3.46 × 10^9^ ± 0.1 × 10^9^ CFU/mL for *P. aeruginosa, E. coli, S. aureus* and *E. faecalis.* Further significant reduction in 140 J (2.5 V for 2760 s) compared to the 60 J (5 V for 300 s) was also detected with *P* of 0.0005, 0.0002, 0.0012, and 0.0002 for *P. aeruginosa, E.coli, S. aureus, and E. faecalis*, respectively. As calculated, the count inhibition percentage recorded 68.72 ± 0.57 and 71.31 ± 0.6% in 60 J groups while it evaluated by 78.62 ± 0.17 and 80.74 ± 0.34% in 140 J groups for *P. aeruginosa,.* For *E. coli,* it recorded 69.75 ± 0.99 and 71.32 ± 0.9% in 60 J groups, while it was 78.91 ± 0.72 and 79.58 ± 0.85% in 140 J groups. Regarding *S. aureus*, 51.30 ± 1.39 and 56.82 ± 1.21% were detected in 60 J groups while 69.78 ± 0.87 and 73.58 ± 0.75% were observed in 140 J groups*;* however, it assessed by 29.49 ± 0.8 and 30.24 ± 1% in 60 J groups, while 47.90 ± 1.8 and 48.20 ± 1.6% were detected in 140 J groups for* E. faecalis.*

**Table 1:** The impact of different electric conditions on bacterial cultivability as evaluated by bacterial count (CFU × 10^9^/mL).

Examined Pathogen	Control	60 J (5 V—300 s)	60 J (2.5 V—1200 s)	140 J (7.5 V—300 s)	140 J (2.5 V—2760 s)
*P. aeruginosa*	5.66 ± 0.33	1.77 ± 0.1	1.69 ± 0.1	1.21 ± 0.07	1.09 ± 0.06
*E. coli*	8.96 ± 0.32	2.71 ± 0.10	2.57 ± 0.09	1.89 ± 0.07	1.83 ± 0.07
*S. aureus*	5.79 ± 0.49	2.82 ± 0.24	2.5 ± 0.21	1.75 ± 0.15	1.53 ± 0.13
*E. faecalis*	6.68 ± 0.18	4.71 ± 0.13	4.66 ± 0.13	3.48 ± 0.1	3.46 ± 0.1

#### Metabolic activity assessment

**Figure 3:**
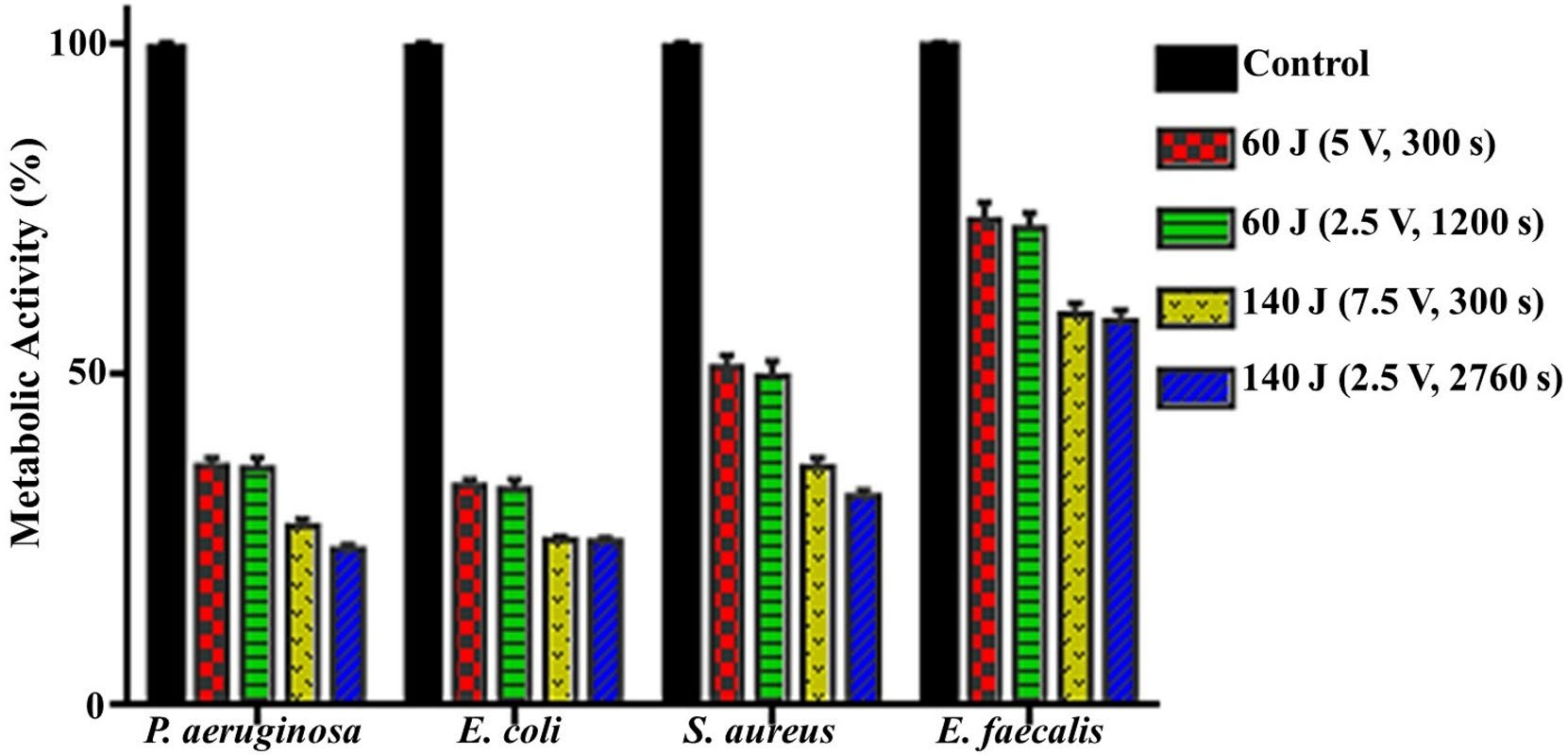
Metabolic performance of examined pathogens after exposure to different electrical conditions compared to control.

The metabolically active cells that survived in a bacteria population could easily metabolize tetrazolium salt to formazan irreversibly. A downward trend was exhibited in the bacterial metabolic activity of examined pathogens upon different treatment strategies, compared to the untreated controls. The metabolic activity percentage is shown in Fig. 3. As calculated, the lowest significant inhibitory impact in metabolic activity was observed in groups of 60 J, in particular that applied at (5 V for 300 s), the inhibition was evaluated by 63.7 ± 1.2, 66.8 ± 1, 48.9 ± 1.9 and 26.6 ± 2.6% for *P. aeruginosa, E. coli, S. aureus* and *E. faecalis*, respectively. On the other hand, superior significant inactivation in cellular metabolism was detected at 140 J (2.5 V for 2760 s), with the recorded activity inhibition of 76.3 ± 0.6, 75.2 ± 0.5, 68.4 ± 1, and 41.9 ± 1.6% for *P. aeruginosa, E. coli, S. aureus,* and *E. faecalis*, respectively.

#### Total and extracellular bacterial protein content

**Figure 4:**
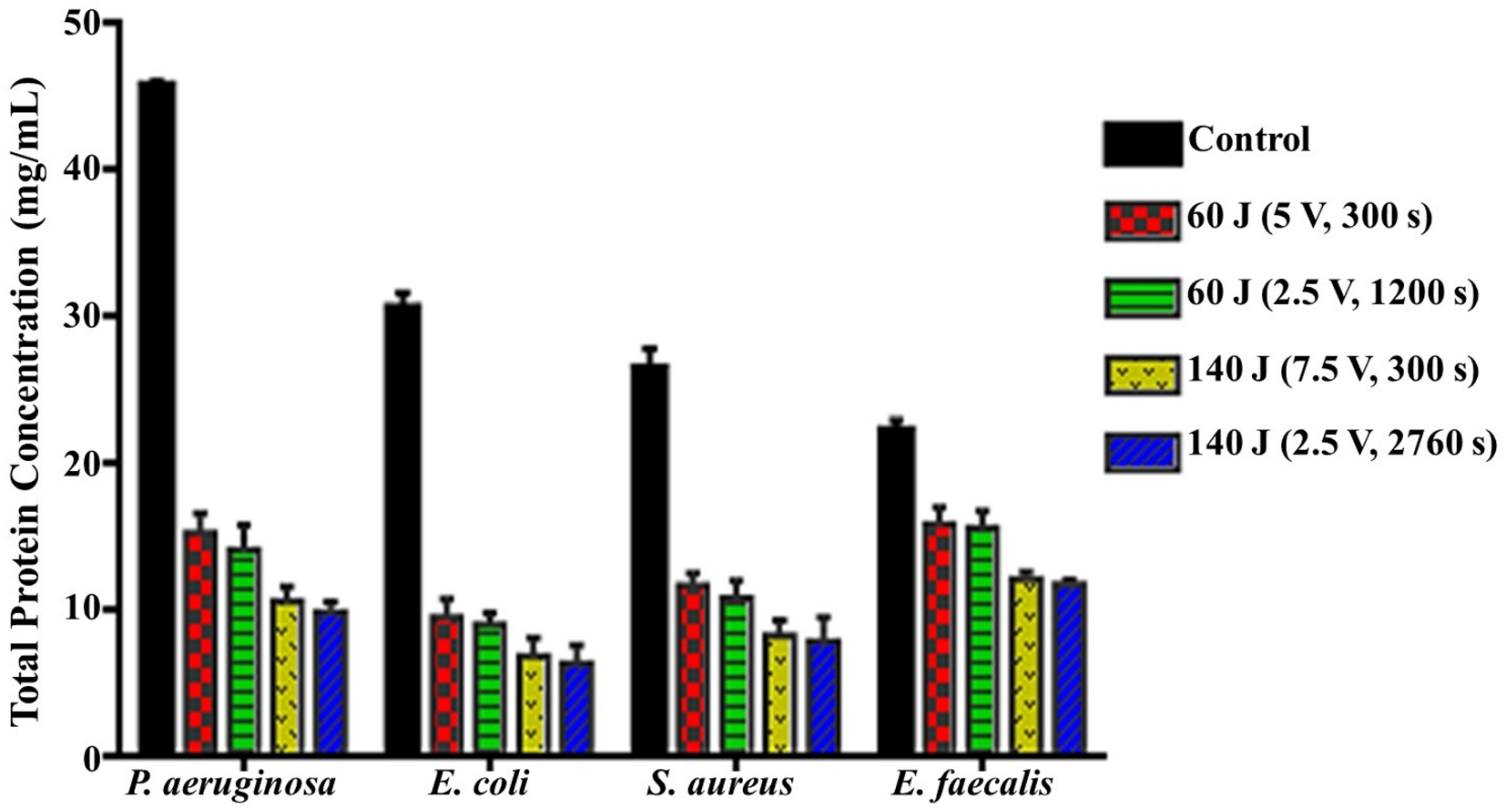
Total protein concentration (mg/mL) after exposure of bacterial strains to different electric conditions compared to control.

Proteins are substantial structural constituents in cellular membranes and cytoplasm, which mediate the main cellular functions including survival, multiplication, and metabolism^20^. Therefore, the total protein concentration could give insinuation about the bacterial functioning level. The leverage of different electrical energy scenarios on the protein content is illustrated in Fig. 4. There was an extremely statistically significant reduction (*P* < 0.0001) in total protein content of *P. aeruginosa, E. coli,* and *S. aureus* exposed to 60 J (5 V for 300 s), compared to control groups, assessed by 66.63 ± 2.91, 68.75 ± 2.67, 55.66% ± 1.28, respectively. While the reduction in total protein content of* E. faecalis* (*P* = 0.001) evaluated by 28.35 ± 1.89%. As its synthesis was suppressed from 45.84 ± 0.28, 30.66 ± 1.02, 26.50 ± 1.38, and 22.33 ± 0.67 mg/mL in untreated control samples and reached 15.30 ± 1.41, 9.58 ± 1.28, 11.75 ± 0.86, and 16.00 ± 1.1 mg/mL in treated bacterial cultures with 60 J (5 V for 300 s), respectively. However, a significant (*P* = 0.0042, 0.0374, 0.0229, and 0.0032) and drastic impact of 140 J (2.5 V for 2760 s) was observed in the total protein content of *P. aeruginosa, E. coli, S. aureus,* and *E. faecalis* compared to 60 J (5 V for 300 s). Their total protein concentration declined to 9.95 ± 0.7, 6.46 ± 1.21, 7.98 ± 1.6, and 11.83 ± 0.3 mg/mL with inhibition percentages reached 78.3 ± 1.4, 78.94 ± 3.23, 69.88 ± 4.99, and 47.04 ± 0.78%, respectively. Similarly, there is no significant change within the same energy groups.

**Figure 5:**
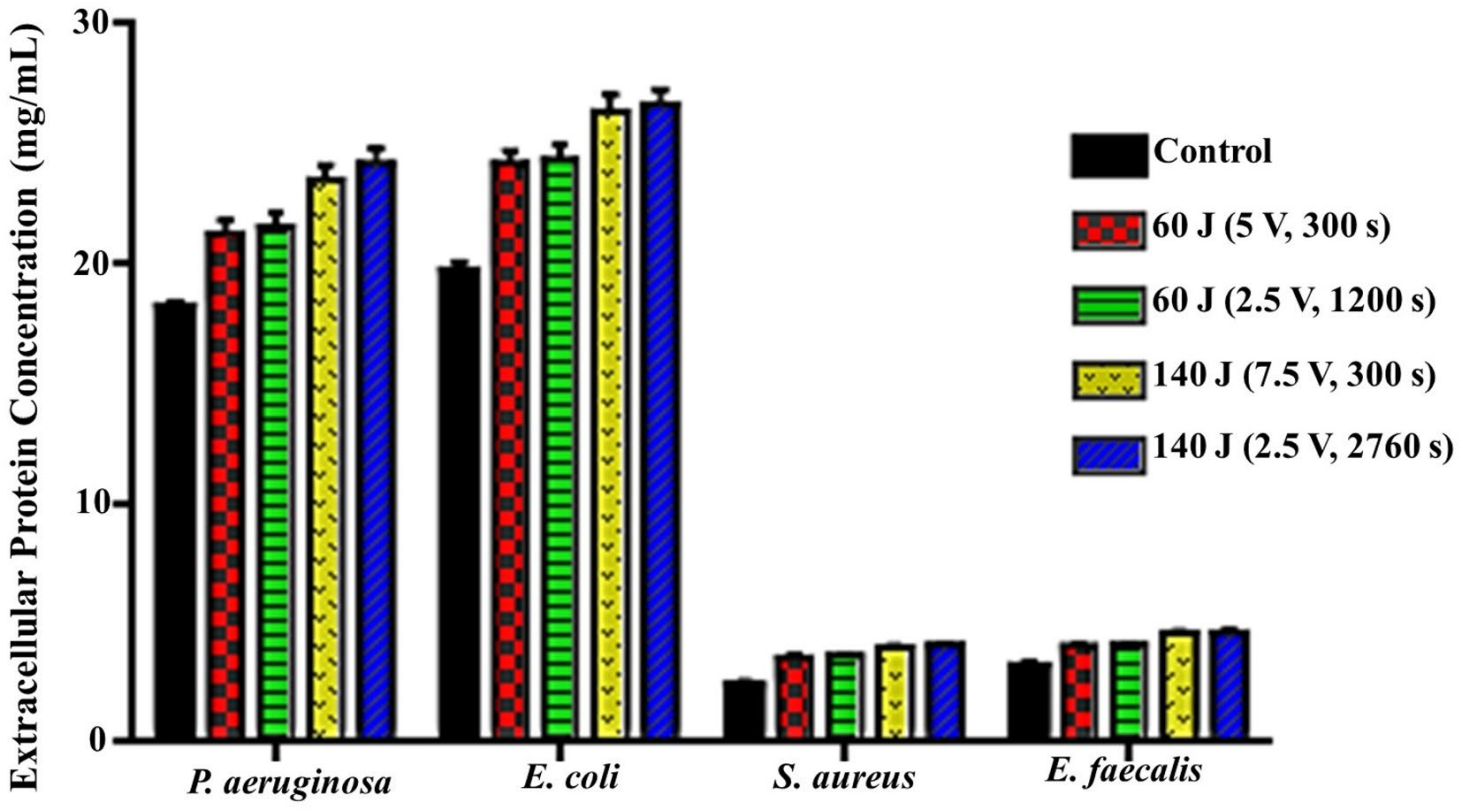
Extracellular protein concentration (mg/mL) after exposure of bacterial strains to different electric conditions compared to control.

Likewise, the results of extracellular protein content shown in Fig. 5 recorded an extremely high significant influence of both applied protocols compared to the control (*P* = 0.0006, 0.0003) for *P. aeruginosa, E. coli,* and (*P* < 0.0001) for *S. aureus* and *E. faecalis*. However, no significance was noticed within the same group and the influence was also in a linear energy dependence. That is clearly evident through the high content of extracellular protein that recorded 21.28 ± 0.5, 24.18 ± 0.55, 3.59 ± 0.06 and 4.07 ± 0.07 mg/mL in treated bacterial cultures upon exposure to 60 J (5 V for 300 s), relative to the untreated control cultures that recorded 18.22 ± 0.22, 19.72 ± 0.35, 2.39 ± 0.07, and 3.18 ± 0.06 mg/mL for *P. aeruginosa, E. coli, S. aureus* and *E. faecalis,* respectively. Remarkably, a significant and more pronounced effect (*P* = 0.0026, 0.0063, 0.0007, and *P* < 0.0001) was detected by employing 140 J (2.5 V for 2760 s). Wherein, the extracellular protein concentration assessed by 24.22 ± 0.57, 26.67 ± 0.61, 4.09 ± 0.07, and 4.61 ± 0.08 mg/mL for *P. aeruginosa, E. coli, S. aureus,* and *E. faecalis,* respectively. Intriguingly, the increase in extracellular protein content of all examined bacterial types upon exposure to different electrical conditions pointed to the cellular damage and cell wall deterioration that resulted in leakage of intracellular content.

#### Effect of different exposure conditions on cell membrane integrity

**Figure 6:**
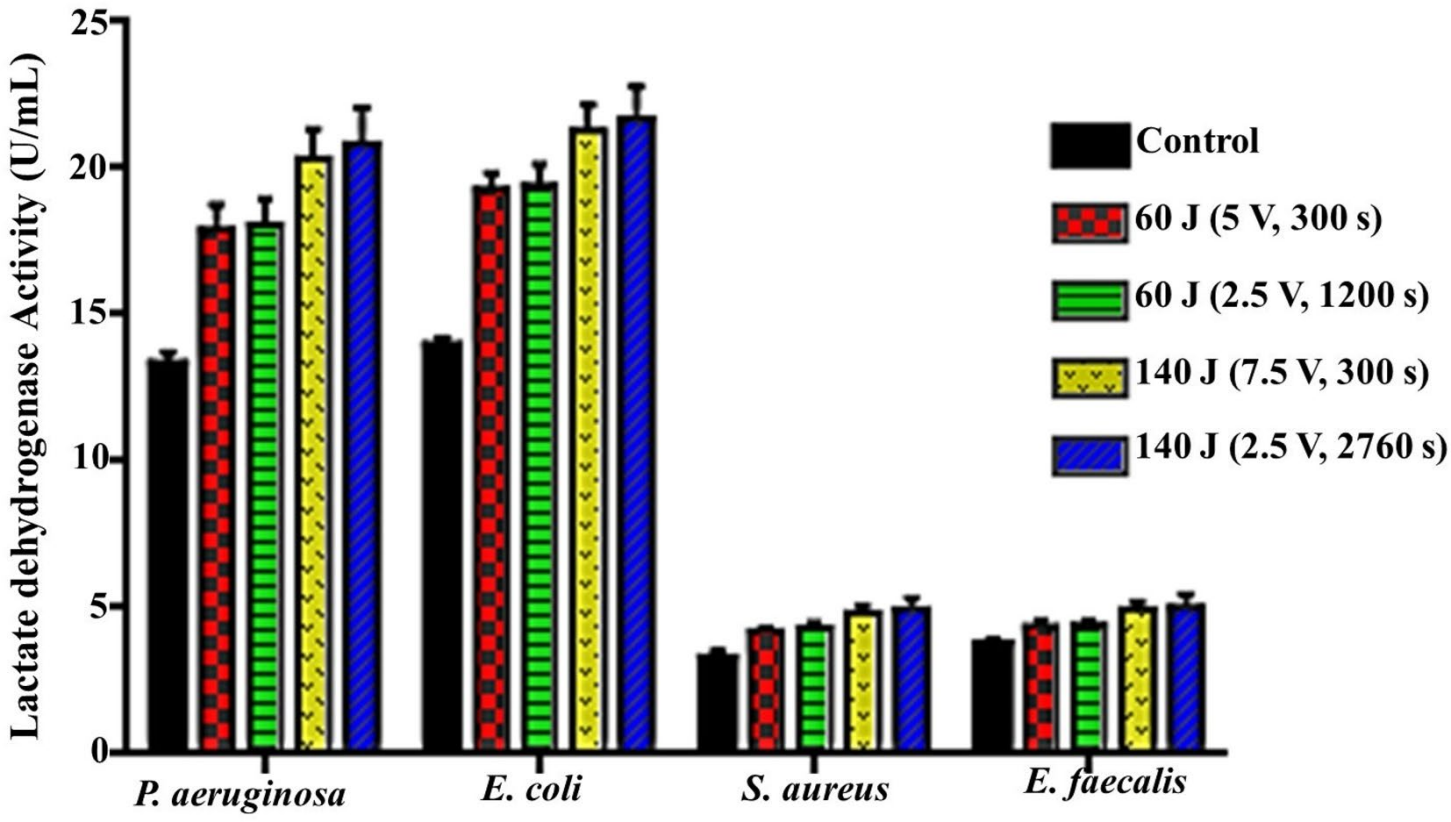
Lactate dehydrogenase activity after exposure of bacterial strains to different electric conditions compared to control.

In the same sense, the lactate dehydrogenase (LDH) activity of all examined bacterial genera was examined to confirm the loss of cell membrane integrity in the damaged cells and the release of their intracellular LDH to the external milieu^25^. The results shown in Fig. 6 emphasized the cytotoxic effect of different DC protocols on the examined pathogens. That is vividly observed through increasing LDH activity compared to the untreated control, which also appeared significant between both major groups (i.e., 60 and 140 J). The average percentage of increment in 60 J groups compared to control was 35.01 ± 0.92%, 38.79 ± 0.61%, 29.73 ± 2.15%, and 16.43 ± 0.29% wherein, LDH activity was assessed by 17.91 ± 0.86, 19.29 ± 0.56, 4.23 ± 0.13, and 4.38 ± 0.22 U/mL in treated bacterial supernatants upon exposure to 60 J (5 V for 300 s), relative to the untreated control that recorded 13.33 ± 0.41, 13.94 ± 0.28, 3.3 ± 0.26, and 3.77 ± 0.18 U/mL (*P* = 0.0011, 0.0001, 0.0052, and 0.0205) for *P. aeruginosa, E. coli, S. aureus,* and *E. faecalis,* respectively. Upon elevating the applied energy to 140 J, a higher average percentage of LDH increased to 54.60 ± 2.72%, 54.29 ± 2.00%, 49.13 ± 3.36%, and 32.92 ± 1.16% compared to the control. By employing 140 J (2.5 V for 2760 s) compared to 60 J (5 V for 300 s), LDH activity significantly raised (*P* = 0.0005, 0.0003, 0.0027, and 0.0104) to 20.86 ± 1.20, 21.71 ± 1.1, 5.00 ± 0.36, and 5.04 ± 0.45 U/mL for *P. aeruginosa, E. coli, S. aureus* and *E. faecalis,* respectively. Despite there being an obvious significance at the inter-group level, no significant change was recorded at the intra-group level.

#### Effect of different exposure conditions on DNA

**Figure 7:**
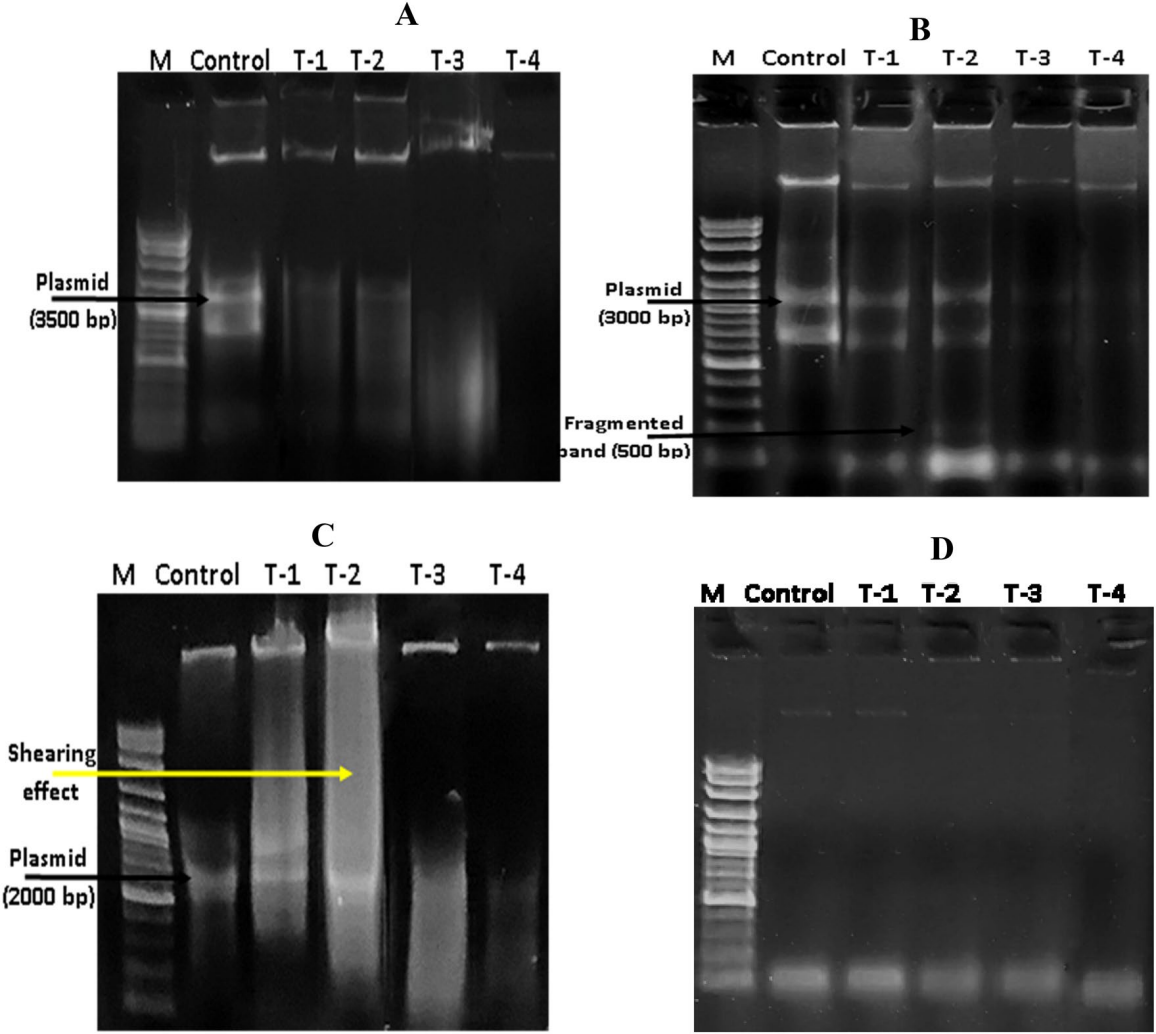
Total genomic DNA after exposure of *P. aeruginosa* (**A**), *E. coli* (**B**), *S. aureus* (**C**) and *E. faecalis* (**D**) to different electric conditions compared to control. Lane (T1) refers to 60 J-applied energy with 5 V for 300 s, Lane (T2) refers to 60 J-applied energy with 2.5 V for 1200 s, Lane (T3) refers to 140 J-applied energy with 7.5 V for 300 s and Lane (T4) refers to 140 J-applied energy with 2.5 V for 2760 s.

**Figure 8:**
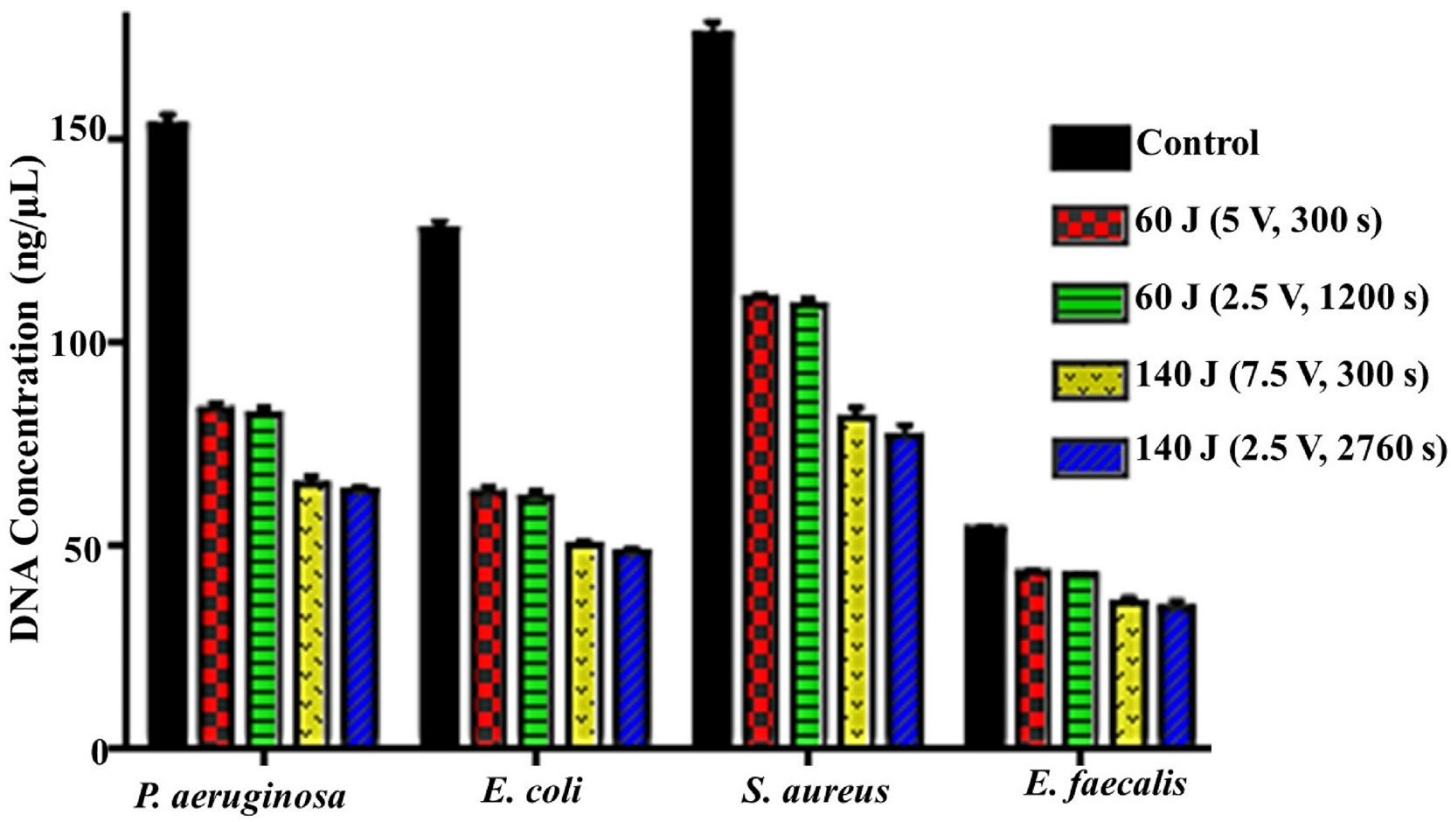
DNA concentration after exposure of bacterial strains to different electrical conditions compared to control.

Despite there was no direct contact with the genetic material of examined pathogens, the genotoxic potential of different DC protocols was clearly evident herein either qualitatively or quantitatively. Figure 5 depicts a distinct decomposability and decline in the DNA band intensity, which is related to their quantity. Let alone the shearing effect of DNA, which is clearly observed in *E. coli* and *S. aureus* (Fig. 7). Additionally, the extra-chromosomal element (i.e., plasmid) was also influenced by different exposure strategies. Wherein, fragmentation of supercoiled structure, shearing and disappearance phenomena were detected in *P. aeruginosa, E. coli* and *S. aureus,* respectively. Interestingly, as shown in Fig. 8, the lowest significant reduction in DNA concentration was detected upon exposure to 60 J (5 V for 300 s), which diminished from 153 ± 3, 127.5 ± 2.5, 175.5 ± 3.5, and 54 ± 1 ng/µL in untreated controls and reached to 84 ± 1.5, 63 ± 1.5, 111 ± 1, and 44 ± 0.5 ng/µL (*P* < 0.0001) for *P. aeruginosa, E. coli, S. aureus*, and *E. faecalis*; signifying by such way a destructive potentiality of 45.42 ± 0.46%, 50.98 ± 0.55%, 37.32 ± 0.81%, and 19.44 ± 1.31%, respectively. However, the highest damage was recorded at 140 J (2.5 V for 2760 s), which significantly lessened the concentration of total genomic material to 64 ± 1, 49 ± 1, 77 ± 2.5, and 35 ± 1.5 ng/µL (*P* < 0.0001, *P* = 0.0001, *P* < 0.0001, and *P* = 0.0007) for *P. aeruginosa, E. coli, S. aureus*, and *E. faecalis,* respectively. Hereby, the destructive potentiality reached 57.84 ± 0.46%, 60.98 ± 0.83%, 54.70 ± 2.01%, and 33.80 ± 1.96%, in the same order. As noticed previously, there were no significant changes between each treatment in the same group.

#### Effect of different exposure conditions on the produced ROS

**Figure 9:**
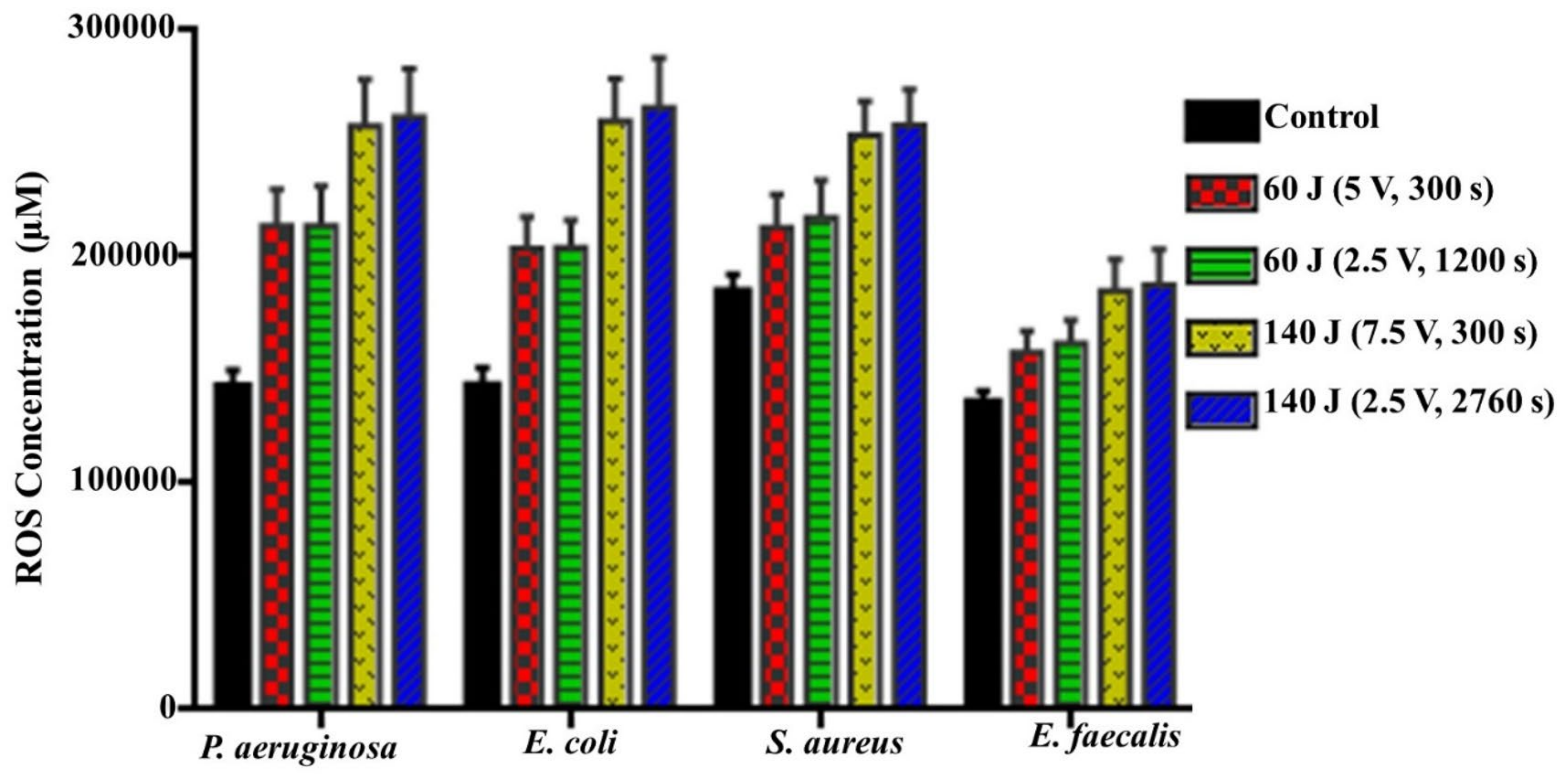
ROS concentration after exposure of bacterial strains to different electrical conditions compared to control.

The stimulation of oxidative stress brought on by the production of ROS as shown in Fig. 9 was identified. As indicated by ANOVA, there is a significant elevation in the produced ROS in 60 J groups compared to the control (*P* = 0.0022, 0.0028, 0.0410, and 0.0222), moreover a noticeable significant (*P* = 0.0358, 0.0291, 0.0216, and 0.0463) increase in ROS generation in 140 J groups compared to 60 J groups. The untreated pathogens generated a low level of ROS during their normal growth cycle and cellular metabolism. However, upon exposure to various electric energy protocols, higher ROS concentrations were developed. As displayed previously, the lowest ROS generation was detected at the strategy of 60 J (5 V for 300 s), which reached to 213,948 ± 16,050, 203,881 ± 13,930, 212,937 ± 14,567, and 158,012 ± 9167 µM, relative to the control that recorded 142,885 ± 7135, 142,902 ± 8153, 184,789.5 ± 7533, and 135,999.5 ± 5134 µM for *P. aeruginosa, E. coli, S. aureus* and *E. faecalis,* respectively. On the other hand, the exposure to 140 J (2.5 V for 2760 s) generated the highest values of ROS by 261,956 ± 21,348, 265,764 ± 22,358, 258,134 ± 15,667, and 187,735 ± 15,550 µM. Arguably, based on the preceding collective results, there is a correlation that seems to be present between different exposure scenarios and their lethal leverage on examined pathogens' viability, metabolic activity, and cellular content.

#### Effect of 140 J DC exposure on bacterial morphology

**Figure 10:**
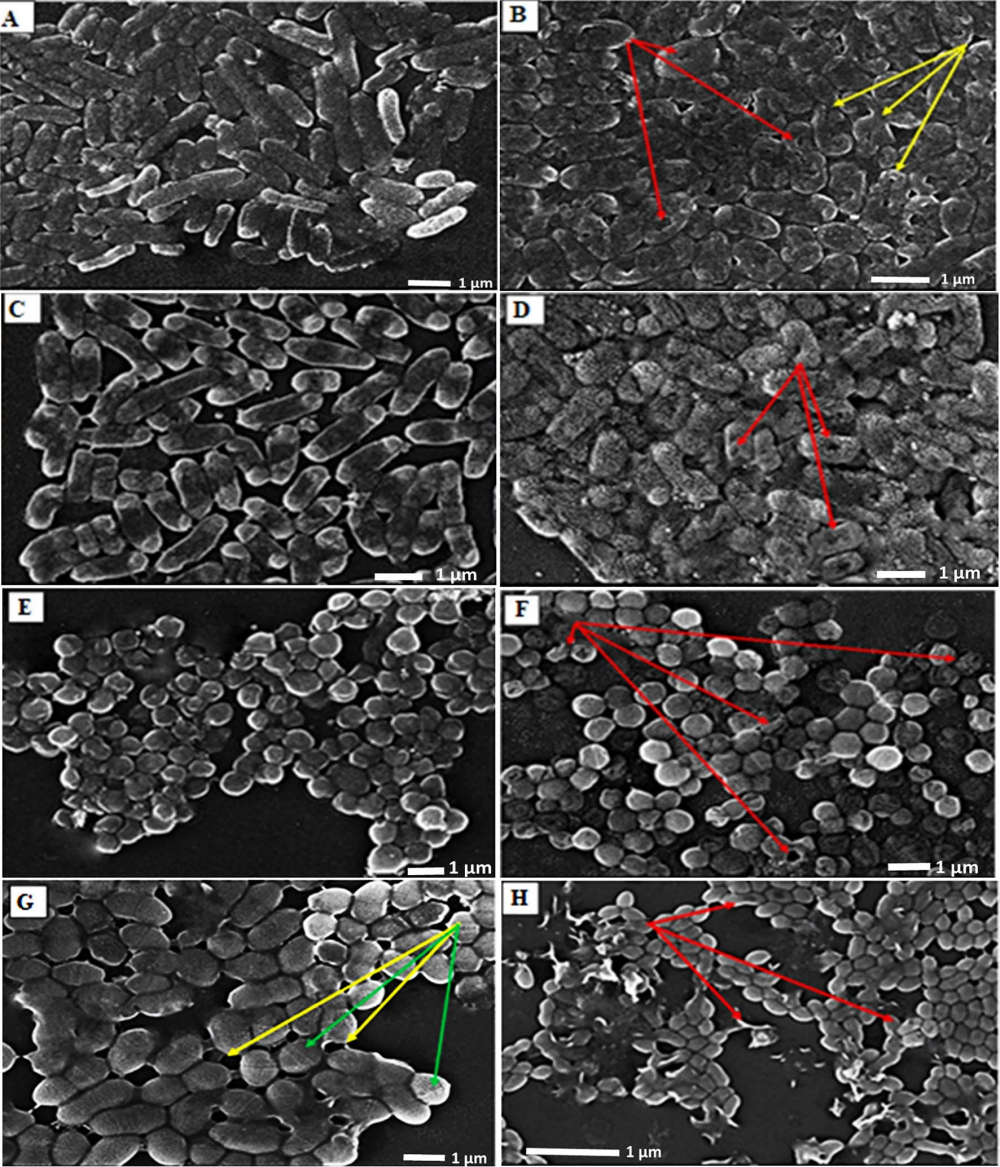
SEM micrographs of control (left panel) and 140 J-treated (right panel) cells of *P. aeruginosa* (**A**,**B**), *E. coli* (**C**,**D**), *S. aureus* (**E**,**F**), and *E. faecalis* (**G**,**H**). Red arrow pointed out to deformed cells with apical crevices furrows and holes. Yellow arrows referred to exopolysaccharides released by the cells; green arrows indicated the position of the septum during cell division. The imaging conditions: SED 20.0 kV, WD 9.7 mm, Std-PC 30.0. HighVac. × 10,000.

To visualize the morphological alterations generated by the most lethal DC strategy (140 J electrical energy with 2.5 V for 2760 s) on the examined bacterial genera, SEM was employed. Generally, the untreated cells appeared healthy and maintained their normal size, shape, and texture; even the septum at mid-cell at the symmetric division stage was clearly observed in the cells of *E. faecalis*, which were embedded in their slimy extracellular polymeric matrix (EPS) (Fig. 8E). Conversely, the electrical treatment brought up obvious deformations in cell shape/surface and shrinkage in size. Such morphological deformations encompassed ruffled cell surfaces, several apical crevices and wide-furrow (Fig. 10 indicated by arrows), which implied cell membranes disruption and further infiltration of cytosolic ingredients extracellularly. Remarkably, cell lysis and disintegration of EPS matrix were detected in *E. faecalis* cells. Thus, SEM micrographs seemed coincident with previous results and emphasized the dramatic effect of DC on the examined bacterial genera.

#### Effect of the most lethal DC condition on bacterial antibiogram pattern

The resistance/sensitivity patterns of the examined pathogens against different antibiotics either before or after the exposure to 140 J electrical energy with 2.5 V for 2760 s are listed in Table 2. As observed the untreated pathogens tolerated more than one antibiotic except *S. aureus*, which seemed to be the most susceptible strain. Whereas, the DC-treatment augmented the potential susceptibility of the pathogens to different antibiotics up to about ninefold, as in the case of Gentamycin with *S. aureus*. The results indicated that the sensitivity of DC-treated *P. aeruginosa* was enhanced against cefepime by 150%, which was the most effective one. However, DC-treatment impaired the most lethal effect of meropenem against *E. coli* cells by 64.2%. In the same sense, the resistance of *S. aureus*, and *E. faecalis* was weakened against erythromycin and ciprofloxacin by 80 and 86.6%, respectively upon exposure to 140 J electrical energy.

**Table 2:** Antibiogram pattern of control and DC-treated pathogens against some antibiotics.

Antibiotic	Control	DC exposed	Antibiotic	Control	DC exposed
	(ZOI/mm)		(ZOI/mm)		
*P. aeruginosa*			*S. aureus*		
Ceftazidine (CAZ)	R	2.3 ± 0.2	Ciprofloxacin (CIP)	1.5 ± 0.1	2.8 ± 0.2
Gentamycin (CN)	R	2.4 ± 0.1	Ofloxacin (OFX)	1.1 ± 0.1	4.7 ± 0.3
Pipadlin (TPZ)	R	2.4 ± 0.1	Gentamycin (CN)	0.5 ± 0.0	4.4 ± 0.2
Ciprofloxacin (CIP)	0.5 ± 0.0	3.1 ± 0.4	Meropenem (MEM)	0.9 ± 0.0	1.8 ± 0.2
Norfloxacin (NOR)	R	2.3 ± 0.2	Rifampin (RD)	0.2 ± 0.0	1.3 ± 0.1
Amikacin (AK)	0.8 ± 0.0	2.6 ± 0.2	Erythromycin (E)	1.4 ± 0.1	3.7 ± 0.3
Cefepime (FEP)	1.2 ± 0.05	3 ± 0.0	Vancomycin (VA)	0.9 ± 0.1	4.2 ± 0.3
Imipenem (IPM)	0.9 ± 0.0	3.6 ± 0.4	Norfloxacin (NOR)	1 ± 0.0	3.6 ± 0.1
*E-coli*			*E. faecalis*		
Ampicilin (AMP)	1.1 ± 0.1	2.8 ± 0.2	Ampicilin (AMP)	1.2 ± 0.1	3.5 ± 0.5
Gentamycin (CN)	R	1.3 ± 0.1	Vancomycin (VA)	0.8 ± 0.0	2.6 ± 0.4
Meropenem (MER)	1.4 ± 0.1	2.3 ± 0.3	Ciprofloxacin (CIP)	R	2.6 ± 0.4
Norfloxacin (NOR)	R	2.1 ± 0.1	Norfloxacin (NOR)	R	2.9 ± 0.6
Ciprofloxacin (CIP)	R	1.5 ± 0.0	Nitrofurantoin (F)	R	2.7 ± 0.3
Ceftazidine (CAZ)	1.8 ± 0.2	3.7 ± 0.3	Rifampin (RD)	1 ± 0.0	3 ± 0.0
Ofloxacin (OFX)	R	1.8 ± 0.1	Erythromycin (E)	1.5 ± 0.0	2.7 ± 0.3
Tigecycline (TGE)	1.1 ± 0.1	2.5 ± 0.2	Doxycycline (DO)	R	2.4 ± 0.1

## Discussion

Microorganisms are definitely omnipresent in earth’s biosphere. Their capacity to decompose organic matter, generate oxygen, supplement nutrients and produce bioactive compounds is accounted to their beneficial functions to preserve the ecosystem in a balanced state. Even in all living creatures within the biological kingdom *Animalia*, they occupy them as commensals or indigenous microbiota. Such micro flora is naturally dwelling animals and human being skin and mucous membranes of multiple cellular systems just after birth until death. They play inevitable roles in metabolism, immunity, cellular modulation and drug interaction^26^. Nonetheless, the pathogenic forms of microbes are serious threaten for health, ecosystem and economy. Pathogenic microbes, which are the main reason for infectious diseases, are ubiquitous and could be easily transmitted through foodborne, waterborne, personal (i.e., fecal/oral from Person-to- Person) and hospital-acquired infection^26,27^. However, *P. aeruginosa, E. coli, S. aureus* and *E. faecalis* are the most popular opportunistic pathogens and are listed among the highly ranked nosocomial pathogens. The endocarditis, bacteremia, urinary tract infections, pneumonia, osteomyelitis, intra-abdominal infections and postsurgical wound infections are the major symptoms of their pathogenicity^28–30^. Therefore, the current study focused on them as paradigms of Gram-negative and Gram-positive pathogens. In recent decades, antibiotic therapy was developed to cease their dissemination and diminish their pathogenicity. Despite the effectiveness of antimicrobial agents and their wide variation of antagonistic mechanisms, their intense and excessive use has led to drug resistance, both within healthcare settings and in the wider community^31^.

Actually, various physicochemical approaches are employed to disinfect water and wastewater. Chlorination, treatment with ultraviolet (UV) and ozonization are the most commonly applied conventional disinfection methods. Despite their remarkable merits, each one suffer from different drawbacks. For chlorination process, chlorine, either gaseous form or the hypochlorite compounds, reacts with naturally-present organic matter (NMO) (e.g., humic and fulvic acids, algal organic matter, wastewater effluent organic matter, etc.) and generates numerous halogenated disinfection byproducts (DBPs) like trihalomethanes(THMs), chlorophenols and haloacetic acids (HAAs), which are recognized by their carcinogenic and mutagenic activities. Generally, the concentration and formation of DBPs strongly be governed by water constituents, residual chlorine and overall operational parameter. Additionally, not all microbial forms are inactivated by chlorine disinfection particularly those spore-forming like *Bacillus* species^32^. On the other hand, disinfection with UV proved its efficiency without using any chemicals or generating disinfection byproducts (DBPs) and without causing pipelines degradation or corrosion. Let alone the simplicity of its operation, no need for storage/transportation tanks and does not significantly change the water quality, thus, no changes in taste or color. However, this approach is not adequate for all types of water or wastewater. Wherein, it suits only clear or pre-filter water without any suspended solids, which will hamper the germicidal effectiveness. Besides, it requires electricity to operate with difficulty in monitoring the UV dose. Notably, UV reactors mostly contain mercury lamps, therefore, any deterioration in UV lamps causes a real mercury hazard^33^. Regarding ozone-disinfection, its complex-operation, corrosive/reactive properties, un-economic procedure with extremely irritating and toxic possibility consider being the major cones of this method^34^. All the previously mentioned limitations concerning the conventional disinfection approaches could possibly restrict their application.

Therefore, trials for efficient disinfectant alternatives have been attracting broad interest and attention^34,35^, including the application of different electric conditions^36,37^ or conjugate low-electrical currents with antibiotics^38–40^. Recently, the application of the electrical field (EF) was employed as a potent disinfection technique to combat pathogenic microbes in water, wastewater, food industry, and medical devices, while retaining the beneficial physicochemical properties of the treated samples^41,42^. However, the high cost associated with extensive energy consumption is the major barrier of EF. According to the literatures, the specific energy consumption of EF for liquid food processing is 40 ~ 1000 kJ/L, assuming the liquid density is 1 kg/L^43,44^. Comparatively, this is significantly higher than that required for some other technologies, which mainly consume electrical energy in water disinfection (e.g., 20 ~ 100 J/L for UV and 20 ~ 150 J/L for ozone)^8,45,46^. Thus, the hybrid energy harvesters, which convert multiple ambient energies simultaneously, deemed as cost-effective alternatives to reduce energy consumption. Recent advancements in hybrid energy systems have been proposed that simultaneously harvest various ambient energies (e.g., photo irradiation, flow kinetic, thermal, and vibration) to drive self-powered water purification processes. These hybrid energy harvesters are based on the mechanisms of mechanical and photovoltaic, mechanical and thermal, and thermal and photovoltaic effects^47^. Hence, the general idea to reduce the energy consumption of EF is to operate the process at lower voltages. When the operating voltage is lower, energy conservation efficiency for pulse generation is typically higher. In addition, side electrochemical reactions and unintentional heating can also be reduced^8^.

In the present study, two electrical energy strategies, generated from DC electric field, were employed as antimicrobial technique. Such scenario was implemented through fluctuating the applied voltage and exposure time. Remarkably, it is plausible to exclude the possibility of bacterial inactivation by thermal effect. Wherein, the increase in temperature owing to the applied electrical energy not exceed 5 °C in both strategies, the overall temperature of the bacterial suspension during and after treatment was in the range of 32–37 °C. This suggestion is consistent with that reported by^48^, who stated that DC electric field (5 V/cm) could be thoroughly inactivate microorganisms in the milk at temperatures below 55 °C.

**Figure 11:**
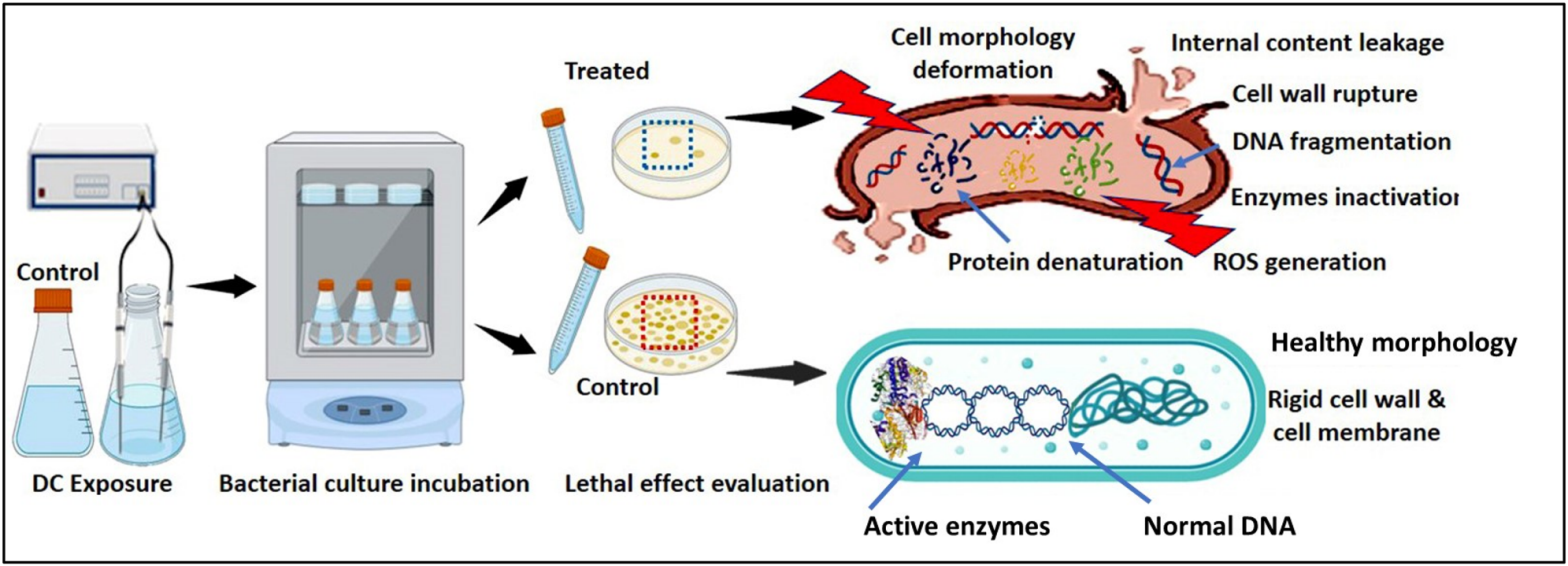
Schematic representation of DC exposure process and subsequent antimicrobial activity evaluation showing DC influence on cell morphology, cell wall, DNA, proteins and ROS generation.

Despite some literatures highlighted the bacteriostatic role of DC^49,50^, others revealed its role in prohibiting microbial growth, lessening their metabolic activity, which ultimately increasing their mortality^51^. This is in agreement with that obtained in our study. Our results revealed the high impact of DC on bacterial growth compared with the control group for all the bacterial strains, and this inhibition increased as the energy increased, while there was no significant change within the same energy groups (at different applied voltages and exposure times). That could be attributed to several decisive parameters as would be explained. Initially, by applying the direct electrical current with different voltage and exposure time, in bacterial suspension under oxygenic condition, generating hereby two electric energy scenarios and subsequently, various chemical oxidants might be produced as a result of electrode electrolysis. Wherein, the bacterial inactivation occurred as a result of these oxidants, especially in the presence of oxygen and other ions in the culture media. As the electrical energy originated from the electrical current increased with a longer duration of treatment or higher electrical potential, such oxidants immediately increased; eventually, the lethal effect became severe. Likewise, Kim et al.^38^ found that a higher reduction in biofilm biomass was conjugated with higher energy supplied either by elongation of exposure time or uplifting the electrical potential regardless of the type of electrical signal. Simultaneously, the scattering of silver ions, from the utilized electrodes to the culture media during exposure may be another factor that participated in such antisepsis. In this sense, Secinti et al.^52^ emphasized the lethal effect of anodic silver. Similarly, Wang et al.^39^ mentioned that the large surface area of the stainless-steel electrodes was responsible for suppressing *P. aeruginosa* cells by the virtue of Fe^2+^, Fe^3+^, Cr^2+^, Cr^3+^, and Cr^6+^ ions that were intensively released during DC treatment. Thus, it is proposed that the bactericidal potentiality of both electrical currents (i.e., 60 J and 140 J) lied behind the synergistic influence of applied energy and also the released silver ions that were dissociated from the electrodes. To confirm this point, the silver ions concentration in each energy group and also the control were measured using ICP. The results unveiled the absence of silver ions in the control, however, they assessed by 16.7 ± 2.16 ppm in 60 J-applied energy with 5 V for 300 s, 41.75 ± 21.85 ppm in 60 J-applied energy with 2.5 V for 1200 s, 122.35 ± 33.02 ppm in 140 J-applied energy with 7.5 V for 300 s and 143.5 ± 30.41 ppm in 140 J-applied energy with 2.5 V for 2760 s, respectively. Therefore, to perceive the inhibition mechanism, which is triggered by both electrical energy protocols, several parameters concerning microbial growth and metabolism were investigated more deeply and comprehensively. In general, there is an obvious lethal concurrence among all examined parameters as represented in schematic graph (Fig. 11).

Wherein, there was a progressive and significant reduction in cultivability, metabolic performance, membrane integrity, total protein content, DNA content and also morphological deformations. On the other hand, there were significant elevation in both extracellular protein content and ROS generation after exposure to both electric energy strategies. In fact, the inhibition percentages calculated from the cultivability test with the range of (68.72 ± 0.57–80.74 ± 0.34%), (69.75 ± 0.99–79.58 ± 0.85%), (51.30 ± 1.39–73.58 ± 0.75%) and (29.49 ± 0.8–48.20 ± 1.6%) for *P. aeruginosa, E. coli, S. aureus,* and *E. faecalis,* respectively, were higher than that recorded in the rest of all examined parameters. While the inhibition in metabolic performance ranged from (63.70 ± 1.2–76.3 ± 0.6%), (66.8 ± 1–75.2 ± 0.5%), (48.9 ± 1.9–68.4 ± 1%), and (26.6 ± 2.6–41.9 ± 1.6%) for *P. aeruginosa, E. coli, S. aureus* and *E. faecalis,* respectively. This finding could be explained by a phenomenon called viable but non-culturable (VBNC) state that was induced by the action of electrical energy. In this case, the bacteria are still alive and exert their physiologically active metabolism for survival but losing their ability to grow and cultivate. Similarly, Xu et al.^20^ reported the same observation for *S. aureus* and *E. coli* after treatment with direct current (DC) air–liquid discharge plasma. Interestingly, Marchal et al.^53^ mentioned that environmental stresses such as UV radiation, energy flow, and hazardous chemicals would trigger the transition of bacteria from the cultivable phase to the VBNC phase. In this context, the LDH enzyme, which expressed the membrane integrity of examined pathogens, was diminished by the range of (35.01 ± 0.92–54.60 ± 2.72%), (38.79 ± 0.61–54.29 ± 2.00%), (29.73 ± 2.15–49.13 ± 3.36%), and (16.43 ± 0.29–32.92 ± 1.16%) for *P. aeruginosa, E. coli, S. aureus,* and *E. faecalis,* respectively; comparing to cultivability, which boosted the results of metabolic performance. Apparently, the applied strategies of electrical energy might be responsible for membrane lipid peroxidation, revisable/irreversible permeability, and membrane puncturing as well. Notably, these results agreed with those found by^54^. Besides, the effect of the electric field on membrane proteins orientation and their electrophoretic mobility were also taken into the account as reasons for weakening membrane integrity^11^. Additionally, the electron transport chain, which is centralized on the bacterial inner membrane and mediates the generation of ATP, would also be deteriorated by the applied electrical energy; eventually blocking the cellular respiration, physiological pathways and cultivability.

Meanwhile, the degradation of bacterial membrane resulted in infiltration of intracellular constituents outside the cells. That was clearly evident through increasing the concentration of extracellular protein after exposure; implying the membrane destruction that followed by cellular leakage. Whereas, the overall protein content in all treatments testified the dramatic decrease, which could be attributed to the intensive effect of electric field on protein alpha-helix structure, subsequently function inactivation^11^. Simultaneously, the quantitative and qualitative gauges of cellular nucleic acid revealed an intelligible reduction in concentration and distortion derived from fragmentation and shearing. Both findings reflect disorder in genomic DNA/ extrachromosomal element configurations, which could generate perturbing in replication, blockage in the DNA-repairing process, prohibition in gene information transmission/ mRNA transcription and readily protein translation errors ended by cell death. Morphologically, as depicted from SEM, several transformations, surface flaws, wide furrows, and condensed cell indentations were detected on cells' surface and the presence of cell debris unveiled the oxidation process induced by the applied electric protocols. Broadly, SEM micrographs mirrored and emphasized the data obtained from the other studied parameters. Likewise,^11^ observed changes in cell shape, dimensions, and cell glycocalyx after exposure to electricity.

Interestingly, the oxidative stress or oxidant injury triggered by the applied electrical conditions and also silver ions contributed substantially to such deleterious effects. The produced ROS exert a considerable affinity toward the main components of bacterial cells, which are organic species including peptidoglycan, lipopolysaccharides, lipids, DNA, RNA, and proteins; thereby, altering the surface morphology, disrupting the cellular structure, inactivating functional moieties, and terminating cellular metabolism^20^. Such signals of ROS impacts on bacterial cells, either planktonic phase or biofilm phase, as a result of DC exposure were stated by other research groups and are consistent with our results^55,56^. Furthermore, based on the collective results of all examined microbial parameters, the Gram-positive bacteria seemed more resistant to differently applied DC strategies. That could be assigned to the polarity and configuration of the Gram-positive bacterial cell wall, which is composed mainly of a thick peptidoglycan layer that acting as a protective exoskeleton against extreme environmental stress^57^. In addition, the examined strains exerted a defensive physiological system to mitigate any environmental stress response. As*, S. aureus* and *E. faecalis* are catalase-positive, which break down hydrogen peroxide into water and oxygen; attenuating thereby the oxidative stress^58,59^. Our finding is matched with^20^.

It is plausible to mention that the inhibitory effect of DC with different energies exerted their lethal effect in a chain of sequenced and synchronized features, which obviously confirmed upon examining the antibiogram potentiality of pathogens after the exposure program. Generally, all treated pathogens became sensitive to several antibiotics, which they resisted in their native-untreated forms. Such results implied the changes in bacterial physiology, metabolic capacity, and resistance mechanisms, which all ended in increasing their antibiotic susceptibility. Subsequently, the present study represents an alternative microbicidal approach to combat microbial pollution in effluents before discharging in water bodies and disseminating epidemic diseases. As a future perspective, the effectiveness of this approach could be enhanced by combining with metals, polymers, carbon-based materials, or even composites in nanostructure. Additionally, the employing of antimicrobial biomolecules such as microbial pigments, surfactants, polymers and phyto-extracts could synergistically promote microbial disinfection in different applications such as wound sterilization, milk sterilization, and wastewater purification.

## Conclusion

The direct current represents a promising microbicidal technology for disinfection and reducing the microbial burden in wounds, food, and wastewater. The direct inactivation impacts of different field energies (i.e., 60 J and 140 J) were employed via different strategies as a function of voltage and exposure time against *P. aeruginosa, E. coli, S. aureus,* and *E. faecalis*. The applied electrical energies imposed significant antibacterial effects on all kinds of microbes relative to the untreated controls. The studied parameters of bacterial cultivability, metabolic performance, membrane integrity, total proteins, extracellular protein content, cellular nucleic acid content, morphology, and ROS indicated bacterial devastation after electricity treatment. Specifically, exposure to 140 J (2.5 V for 2760 s) generated the maximum and the highest significant inhibition in bacterial counts by percentages calculated as 80.74 ± 0.34, 79.58 ± 0.85, 73.58 ± 0.75, and 48.20 ± 1.6% for *P. aeruginosa, E. coli, S. aureus* and *E. faecalis,* respectively. Generally, Gram-positive bacteria displayed less susceptibility against the applied electrical strategies than Gram-negative bacteria. Besides, declining tendencies was observed in all examined cellular components but lower than that detected in the bacterial count; reflecting the lethal effect of electricity and the possibility of inducing a physiologically viable but non-culturable (VBNC) state. Finally, the exposure to DC with different energies enhanced the antibiotic sensitivity against different antibiotics, which symbolizes a main key to defeating pathogens in conjugation with other therapies in prospective studies.

## Data Availability

All data generated or analyzed during this study are included in this published article.

## References

[1] Jannesari, M. *et al.* Boosting on-demand antibacterial activity using electrical stimulations from polypyrrole-graphene oxide triboelectric nanogenerator. *Nano Energy* **1**, 108463 (2023). 10.1016/j.nanoen.2023.108463

[2] Wang, C. H., Hsieh, Y. H., Powers, Z. M. & Kao, C. Y. Defeating antibiotic-resistant bacteria: Exploring alternative therapies for a post-antibiotic era. *Int. J. Mol. Sci.* **5**, 1–18 (2020). 10.3390/ijms21031061 PMC7037027 32033477

[3] Nji, E. *et al.* High prevalence of antibiotic resistance in commensal Escherichia coli from healthy human sources in community settings. *Sci. Rep.* **11**, 1–11 (2021). 33564047 10.1038/s41598-021-82693-4 PMC7873077

[4] Tang, J. *et al.* Research progress of electrochemical oxidation and self-action of electric field for medical wastewater treatment. *Front. Microbiol.* **6**, 1083974 (2023). 10.3389/fmicb.2022.1083974 PMC9853389 36687586

[5] Smith, M. *Antibiotic Resistance Mechanisms* 95–99 (World Scientific, 2017).

[6] Shuai, C. *et al.* strawberry-like Ag-decorated barium titanate enhances piezoelectric and antibacterial activities of polymer scaffold. *Nano Energy* **1**, 104825 (2020). 10.1016/j.nanoen.2020.104825

[7] Zhou, J., Hung, C. Y. & Xie, X. Application of electric field treatment (EFT) for microbial control in water and liquid food. *J. Hazard. Mater.* **445**, 130561 (2023). 37055970 10.1016/j.jhazmat.2022.130561

[8] Zhou, J., Hung, C. Y. & Xie, X. Making waves: Pathogen inactivation by electric field treatment: From liquid food to drinking water. *Water Res.* **207**, 117817 (2021). 34763276 10.1016/j.watres.2021.117817

[9] Sultana, S. T., Babauta, J. T. & Beyenal, H. Electrochemical biofilm control: A review. *Biofouling.* **31**, 745–758 (2015). 26592420 10.1080/08927014.2015.1105222 PMC4668948

[10] Asadi, M. R. & Torkaman, G. Bacterial inhibition by electrical stimulation. *Adv. Wound Care* **3**, 91–97 (2014). 10.1089/wound.2012.0410 PMC3929208 24761349

[11] Del Pozo, J. L., Rouse, M. S. & Patel, R. Bioelectric effect and bacterial biofilms. A systematic review. *Int. J. Artif. Organs.* **31**, 786–795 (2008). 18924090 10.1177/039139880803100906 PMC3910516

[12] Wong, W. F. & Santiago, M. Microbial approaches for targeting antibiotic-resistant bacteria. *Microb. Biotechnol.* **10**, 1047–1053 (2017). 28771951 10.1111/1751-7915.12783 PMC5609231

[13] Hari, P., Kacharaju, K. R., Anumala, N., Pathakota, K. R. & Avula, J. Application of bioelectric effect to reduce the antibiotic resistance of subgingival plaque biofilm: An in vitro study. *J. Indian Soc. Periodontol.* **22**, 133–139 (2018). 29769768 10.4103/jisp.jisp_320_17 PMC5939021

[14] Mo, F. *et al.* Decoupling locally enhanced electric field treatment (LEEFT) intensity and copper release by applying asymmetric electric pulses for water disinfection. *Water Res.* **21**, 100206 (2023). 10.1016/j.wroa.2023.100206 PMC10719566 38098885

[15] Zaki, S. A., Eltarahony, M. M. & Abd-El-Haleem, D. A. Disinfection of water and wastewater by biosynthesized magnetite and zerovalent iron nanoparticles via NAP-NAR enzymes of Proteus mirabilis 10B. *Environ. Sci. Pollut. Res.* **1**, 23661–23678 (2019). 10.1007/s11356-019-05479-2 31201708

[16] El-Naggar, N. E., Dalal, S. R., Zweil, A. M. & Eltarahony, M. Artificial intelligence-based optimization for chitosan nanoparticles biosynthesis, characterization and in-vitro assessment of its anti-biofilm potentiality. *Sci. Rep.* **16**, 4401 (2023). 10.1038/s41598-023-30911-6 PMC10019797 36928367

[17] Bradford, M. M. A rapid and sensitive method for the quantitation of microgram quantities of protein utilizing the principle of protein-dye binding. *Anal Biochem.* **72**, 248–254 (1976). 942051 10.1016/0003-2697(76)90527-3

[18] Arokiyaraj, S. *et al.* Rapid green synthesis of silver nanoparticles from Chrysanthemum indicum L. and its antibacterial and cytotoxic effects: An in vitro study. *Int. J. Nanomed.* **9**, 379–388 (2014). 10.2147/IJN.S53546 PMC3890422 24426782

[19] Elrashdy, R. & Abd-El-Haleem, D. Molecular analysis of cross-bacterial contamination detected in biotin-free buffers during diagnosis of HCV infections. *J. App. Sci. Environ. Manag.* **9**, 5–10 (2005).

[20] Xu, Z. *et al.* In vitro antimicrobial effects and mechanisms of direct current air-liquid discharge plasma on planktonic Staphylococcus aureus and Escherichia coli in liquids. *Bioelectrochemistry* **1**, 125–134 (2018). 10.1016/j.bioelechem.2018.01.012 29413862

[21] Abu-Serie, M. M. Targeted ferroptotic potency of ferrous oxide nanoparticles-diethyldithiocarbamate nanocomplex on the metastatic liver cancer. *Front. Pharmacol.* **13**, 1089667 (2023). 36686682 10.3389/fphar.2022.1089667 PMC9847675

[22] Kirby, W. M. M., Yoshihara, G. M., Sundsted, K. S. & Warren, J. H. Clinical usefulness of a single disc method for antibiotic sensitivity testing. *Antibiotics Annu.* **1957**, 892 (1957). 13425478

[23] Jorgensen, J. H. & Turnidge, J. D. *Susceptibility Test Methods: Dilution and Disk Diffusion. methods Manual of Clinical Microbiology* 9th edn, 1153–1173 (ASM Press, 2015).

[24] Girmay, W. *et al.* Isolation and identification of methicillin-resistant Staphylococcus aureus (MRSA) from milk in shire dairy farms, Tigray, Ethiopia. *Vet. Med. Int.* **2020**, 1–7 (2020). 10.1155/2020/8833973 PMC7443710 32864088

[25] Van den Bossche, S., Vandeplassche, E., Ostyn, L., Coenye, T. & Crabbé, A. Bacterial interference with lactate dehydrogenase assay leads to an underestimation of cytotoxicity. *Front. Cell. Infect. Microbiol.* **10**, 494 (2020). 33042868 10.3389/fcimb.2020.00494 PMC7523407

[26] Balali, G. I., Yar, D. D., Afua Dela, V. G. & Adjei-Kusi, P. Microbial contamination, an increasing threat to the consumption of fresh fruits and vegetables in today’s world. *Int. J. Microbiol.* **2020**, 1–13 (2020). 10.1155/2020/3029295 PMC7269610 32565813

[27] Matinyi, S. *et al.* Contamination of microbial pathogens and their antimicrobial pattern in operating theatres of peri-urban eastern Uganda: A cross-sectional study. *BMC Infect. Dis.* **18**, 1–9 (2018). 30200891 10.1186/s12879-018-3374-4 PMC6131813

[28] Selleck, E. M., Van Tyne, D. & Gilmore, M. S. Pathogenicity of enterococci. *Microbiol. Spectr.* **7**, 53 (2019). 10.1128/microbiolspec.gpp3-0053-2018 PMC6629438 31298205

[29] Cheung, G. Y., Bae, J. S. & Otto, M. Pathogenicity and virulence of *Staphylococcus aureus*. *Virulence* **31**, 547–569 (2021). 10.1080/21505594.2021.1878688 PMC7872022 33522395

[30] Pavlović, M. G., Pavlović, M. M., Pavlović, M. & Nikolić, N. D. Electrochemical removal of microorganisms in drinking water. *Int. J. Electrochem. Sci.* **1**, 8249–8262 (2014). 10.1016/S1452-3981(23)11044-3

[31] Cao, Y. *et al.* Non- antibiotic antimicrobial agents to combat biofilm-forming bacteria. *J. Glob. Antimicrob. Resist.* **21**, 445–451 (2020). 31830536 10.1016/j.jgar.2019.11.012

[32] Al-Abri, M. *et al.* Chlorination disadvantages and alternative routes for biofouling control in reverse osmosis desalination. *NPJ Clean Water.* **2**(1), 2 (2019). 10.1038/s41545-018-0024-8

[33] Paidalwar, A. A. & Khedikar, I. P. Overview of water disinfection by UV technology: A review. *Int. J. Sci. Technol. Eng.* **2**(9), 213–219 (2016).

[34] François, B., Jafri, H. S. & Bonten, M. Alternatives to antibiotics. *Intensive Care Med.* **42**, 2034–2036 (2016). 27033888 10.1007/s00134-016-4339-y

[35] Allen, H. K., Trachsel, J., Looft, T. & Casey, T. A. Finding alternatives to antibiotics. *Ann. N. Y. Acad. Sci.* **1323**, 91–100 (2014). 24953233 10.1111/nyas.12468

[36] Shawki, M. M. & Gaballah, A. The effect of low AC electric field on bacterial cell death. *Roman. J. Biophys* **25**, 163–172 (2015).

[37] Shawki, M. M., Eltarahony, M. M. & Moustafa, M. E. Combined effect of zinc oxide nanoparticles and low electric field in growth suppression of some free-living pathogens. *Curr. Nanosci.* **18**(4), 535–544 (2022). 10.2174/1573413717666211026151538

[38] Kim, Y. W. *et al.* Effect of electrical energy onthe efficacy of biofilm treatment using the bioelectric effect. *npj Biofilms Microbiom.* **15016**, 1–8 (2015). 10.1038/npjbiofilms.2015.16 PMC5515217 28721233

[39] Wang, W. *et al.* Antibiotic resistance: A rundown of a global crisis. *Infect. Drug Resist.* **11**, 1645–1658 (2018). 30349322 10.2147/IDR.S173867 PMC6188119

[40] Wang, H. & Ren, D. Controlling Streptococcus mutans and Staphylococcus aureus biofilms with direct current and chlorhexidine. *AMB Express* **7**(1), 1–9 (2017). 29143221 10.1186/s13568-017-0505-z PMC5688048

[41] Król, Ż, Marycz, K., Kulig, D., Marędziak, M. & Jarmoluk, A. Cytotoxicity, bactericidal, and antioxidant activity of sodium alginate hydrosols treated with direct electric current. *Int. J. Mol. Sci.* **18**(3), 6782017 (2017). 10.3390/ijms18030678 PMC5372688 28327520

[42] Ghernaout, D. Electric field (EF) in the core of the electrochemical (EC) disinfection. *Open Access Lib. J.* **7**(7), 1–20 (2020).

[43] Timmermans, R. *et al.* Pulsed electric field processing of different fruit juices: Impact of pH and temperature on inactivation of spoilage and pathogenic micro-organisms. *Int. J. Food Microbiol.* **173**, 105–111 (2014). 24418831 10.1016/j.ijfoodmicro.2013.12.022

[44] Walter, L., Knight, G., Ng, S. Y. & Buckow, R. Kinetic models for pulsed electric field and thermal inactivation of Escherichia coli and Pseudomonas fluorescens in whole milk. *Int. Dairy J.* **57**, 7–14 (2016). 10.1016/j.idairyj.2016.01.027

[45] Chang, Y. *et al.**Evaluation of Dynamic Energy Consumption of Advanced Water and Wastewater Treatment Technologies* (AWWA Research Foundation & California Energy Commission, 2008).

[46] Huo, Z. Y. *et al.* A Cu3P nanowire enabling high-efficiency, reliable, and energy-efficient low-voltage 338 electroporation-inactivation of pathogens in water. *J. Mater. Chem. A* **6**(39), 18813–18820 (2018). 10.1039/C8TA06304D

[47] Huo, Z. *et al.* Hybrid energy harvesting systems for self-powered sustainable water purification by harnessing ambient energy. *Front. Environ. Sci. Eng.* **17**(10), 118 (2023). 37096021 10.1007/s11783-023-1718-9 PMC10115484

[48] Ji, F., Sun, J., Sui, Y., Qi, X. & Mao, X. Microbial inactivation of milk by low intensity direct current electric field: Inactivation kinetics model and milk characterization. *Curr. Res. Food Sci.* **1**(5), 1906–1915 (2022). 10.1016/j.crfs.2022.10.015 PMC9589170 36300164

[49] Ashrafi, M., Baguneid, M., Alonso-Rasgado, T., Rautemaa-Richardson, R. & Bayat, A. Cutaneous wound biofilm and the potential for electrical stimulation in management of the microbiome. *Future Microbiol.* **12**(4), 337–357 (2017). 28287302 10.2217/fmb-2016-0204

[50] Petrofsky, J., Laymon, M., Chung, W., Collins, K. & Yang, T. N. Effect of electrical stimulation on bacterial growth. *J. Orthop. Neurolsurg.* **31**, 43 (2008).

[51] Czerwińska-Główka, D. & Krukiewicz, K. A. Journey in the complex interactions between electrochemistry and bacteriology: From electroactivity to electromodulation of bacterial biofilms. *Bioelectrochemistry* **131**, 1–14 (2020). 10.1016/j.bioelechem.2019.107401 31707278

[52] Secinti, K. D. *et al.* Antibacterial effects of electrically activated vertebral implants. *J. Clin. Neurosci.* **15**(4), 434–9 (2008). 18281219 10.1016/j.jocn.2007.03.010

[53] Marchal, F. *et al.* Inactivation of Gram-positive biofilms by low-temperature plasma jet at atmospheric pressure. *J. Phys. D* **45**(34), 345202 (2012). 10.1088/0022-3727/45/34/345202

[54] Luo, Q., Wang, H., Zhang, X. & Qian, Y. Effect of direct electric current on the cell surface properties of phenol-degrading bacteria. *Appl. Environ. Microbiol.* **71**(1), 423–427 (2005). 15640217 10.1128/AEM.71.1.423-427.2005 PMC544265

[55] Ezraty, B., Gennaris, A., Barras, F. & Collet, J. F. Oxidative stress, protein damage and repair in bacteria. *Nat. Rev. Microbiol.* **15**, 385–396 (2017). 28420885 10.1038/nrmicro.2017.26

[56] Zou, P., Cao, P., Liu, J., Li, P. & Luan, Q. Comparisons of the killing effect of direct current partially mediated by reactive oxygen species on Porphyromonas gingivalis and Prevotella intermedia in planktonic state and biofilm state–an in vitro study. *J. Dent. Sci.* **17**(1), 459–67 (2022). 35028071 10.1016/j.jds.2021.07.025 PMC8739843

[57] Garde, S., Chodisetti, P. K. & Reddy, M. Peptidoglycan: Structure, synthesis, and regulation. *EcoSal Plus* **15**, 9 (2021). 10.1128/ecosalplus.ESP-0010-2020 PMC11168573 33470191

[58] Mustafa, H. S. *Staphylococcus aureus* can produce catalase enzyme when adding to human WBCs as a source of H_2_O_2_ productions in human plasma or serum in the laboratory. *Open J. Med. Microbiol.* **4**(04), 249 (2014). 10.4236/ojmm.2014.44028

[59] Baureder, M., Reimann, R. & Hederstedt, L. Contribution of catalase to hydrogen peroxide resistance in Enterococcus faecalis. *FEMS Microbiol. Lett.* **331**(2), 160–164 (2012). 22486165 10.1111/j.1574-6968.2012.02567.x

